# Influence of wood species on toxicity of log-wood stove combustion aerosols: a parallel animal and air-liquid interface cell exposure study on spruce and pine smoke

**DOI:** 10.1186/s12989-020-00355-1

**Published:** 2020-06-15

**Authors:** Tuukka Ihantola, Sebastiano Di Bucchianico, Mikko Happo, Mika Ihalainen, Oskari Uski, Stefanie Bauer, Kari Kuuspalo, Olli Sippula, Jarkko Tissari, Sebastian Oeder, Anni Hartikainen, Teemu J. Rönkkö, Maria-Viola Martikainen, Kati Huttunen, Petra Vartiainen, Heikki Suhonen, Miika Kortelainen, Heikki Lamberg, Ari Leskinen, Martin Sklorz, Bernhard Michalke, Marco Dilger, Carsten Weiss, Gunnar Dittmar, Johannes Beckers, Martin Irmler, Jeroen Buters, Joana Candeias, Hendryk Czech, Pasi Yli-Pirilä, Gülcin Abbaszade, Gert Jakobi, Jürgen Orasche, Jürgen Schnelle-Kreis, Tamara Kanashova, Erwin Karg, Thorsten Streibel, Johannes Passig, Henri Hakkarainen, Jorma Jokiniemi, Ralf Zimmermann, Maija-Riitta Hirvonen, Pasi I. Jalava

**Affiliations:** 1 Department of Environmental and Biological Sciences, University of Eastern Finland, Yliopistonranta 1, P.O.Box 1627, FI-70210 Kuopio, Finland; 2 Joint Mass Spectrometry Center (JMSC) at Comprehensive Molecular Analytics (CMA), Helmholtz Zentrum München, Ingolstädter Landstraße 1, D-85764 Neuherberg, Germany; 3 Ramboll Finland, P.O.Box 25 Itsehallintokuja 3, FI-02601 Espoo, Finland; 4 Present address: Savonia University of applied sciences, Microkatu 1, FI-70210 Kuopio, Finland; 5 Finnish Meteorological Institute, Yliopistonranta 1 F, FI-70210 Kuopio, Finland; 6 Joint Mass Spectrometry Center (JMSC) at Analytical Chemistry, Institute of Chemistry, University of Rostock, Dr. Lorenzweg 2, D-18051 Rostock, Germany; 7 Research Unit Analytical BioGeoChemistry, Helmholtz Zentrum München, Ingolstädter Landstraße 1, D-85764 Neuherberg, Germany; 8 Institute of Toxicology and Genetics, Karlsruhe Institute of Technology, Campus North, D-76344 Eggenstein-Leopoldshafen, Germany; 9 Luxembourg institute of health, 1A-B rue Thomas Edison, 1445 Strassen, Luxembourg; 10 Institute of Experimental Genetics (IEG), Helmholtz Zentrum München, Ingolstädter Landstraße 1, D-85764 Neuherberg, Germany; 11 Technical University of Munich, Chair of Experimental Genetics, D-85350 Freising-Weihenstephan, Germany; 12 German Center for Diabetes Research (DZD), D-85764 Neuherberg, Germany; 13 ZAUM - Center of Allergy & Environment, Technical University Munich/Helmholtz Center Munich, Biedersteiner Str. 29, D-80802 Munich, Germany; 14 Max-Delbrück-Centrum für Molekulare Medizin (MDC), Robert-Rössle-Str. 10, D-13125 Berlin, Germany

**Keywords:** Particulate matter (PM), Air liquid-interface (ALI), Inhalation toxicology, Wood combustion, Transcriptome, proteome, Genotoxicity

## Abstract

**Background:**

Wood combustion emissions have been studied previously either by in vitro or in vivo models using collected particles, yet most studies have neglected gaseous compounds. Furthermore, a more accurate and holistic view of the toxicity of aerosols can be gained with parallel in vitro and in vivo studies using direct exposure methods. Moreover, modern exposure techniques such as air-liquid interface (ALI) exposures enable better assessment of the toxicity of the applied aerosols than, for example, the previous state-of-the-art submerged cell exposure techniques.

**Methods:**

We used three different ALI exposure systems in parallel to study the toxicological effects of spruce and pine combustion emissions in human alveolar epithelial (A549) and murine macrophage (RAW264.7) cell lines. A whole-body mouse inhalation system was also used to expose C57BL/6 J mice to aerosol emissions. Moreover, gaseous and particulate fractions were studied separately in one of the cell exposure systems. After exposure, the cells and animals were measured for various parameters of cytotoxicity, inflammation, genotoxicity, transcriptome and proteome.

**Results:**

We found that diluted (1:15) exposure pine combustion emissions (PM_1_ mass 7.7 ± 6.5 mg m^− 3^, 41 mg MJ^− 1^) contained, on average, more PM and polycyclic aromatic hydrocarbons (PAHs) than spruce (PM_1_ mass 4.3 ± 5.1 mg m^− 3^, 26 mg MJ^− 1^) emissions, which instead showed a higher concentration of inorganic metals in the emission aerosol. Both A549 cells and mice exposed to these emissions showed low levels of inflammation but significantly increased genotoxicity. Gaseous emission compounds produced similar genotoxicity and a higher inflammatory response than the corresponding complete combustion emission in A549 cells. Systems biology approaches supported the findings, but we detected differing responses between in vivo and in vitro experiments.

**Conclusions:**

Comprehensive in vitro and in vivo exposure studies with emission characterization and systems biology approaches revealed further information on the effects of combustion aerosol toxicity than could be achieved with either method alone. Interestingly, in vitro and in vivo exposures showed the opposite order of the highest DNA damage. In vitro measurements also indicated that the gaseous fraction of emission aerosols may be more important in causing adverse toxicological effects. Combustion aerosols of different wood species result in mild but aerosol specific in vitro and in vivo effects.

## Highlights

- We studied the toxic effects of wood combustion aerosols.
- Cytotoxic, inflammatory, genotoxic and omics assays were conducted.
- Mild inflammation and significant genotoxicity were detected in both cells and mice.
- Multi-omics approach provided significant new information.

## Background

Globally, the combustion of solid fuels produces high levels of outdoor and indoor pollutants, making it one of the leading causes of premature deaths [1]. According to Silva et al. [2], pollutants from residential and commercial sectors contribute to almost one-third of the premature deaths caused by environmental factors with up to 4.2 million deaths in 2015 associated with long-term exposure to fine particulate matter (PM_2.5_) [1]. PM pollution could become even more severe in the future, as uncontrollably high amounts of PM are released by escalating cases of wildfires (bushfires) [3].

In Europe, emissions from small-scale wood combustion appliances are the most prevalent in winter, and the daily average limits of PM air pollution are exceeded in many regions according to EU directive 2005/50/EC. These violations of limit values are partly due to poor mixing in the atmosphere and the use of old furnaces in household heating [4]. Furthermore, European wood-based residential heating is estimated to double in the future, whereas total fossil fuel usage is projected to decrease [4, 5]. In Europe, spruce and pine are widely used fuel sources for residential heating furnace combustion. For example, in Finland, the usage is estimated to represents 35% of all wood fuels between 2016 and 2017 (Torvelainen [6]). Several studies have investigated the effect of fuel on emissions. However, there are still large gaps in knowledge concerning the toxicity of biomass combustion emissions [7–12]. The composition of biomass combustion emissions depends heavily on combustion efficiency as a result of combustion technology, fuel type, and user practices, which all greatly affect emissions [4, 5, 7, 13–18].

Epidemiological studies have associated exposure to combustion-derived PM with an increased incidence of several diseases, such as chronic obstructive pulmonary disease (COPD), cardiovascular diseases, stroke, Alzheimer’s disease, and cancer [19–22]. However, these investigations are not designed to elucidate the mechanisms of toxicity caused by differing PM compositions. In vivo models are necessary to reveal the pulmonary, cardiovascular, and neurological effects of either complete aerosols or PM collected from combustion emissions [23]. In vitro models have been used to reveal the mechanisms of PM toxicity [14, 24, 25]. To study airborne exposure directly, without the need for collecting particulate samples or extracting them from filters, new solutions for inhalation toxicology using air liquid interphase (ALI) systems have been developed [26–28]. In ALI exposures, the toxicological effects of particulate and gaseous phases of emissions can be assessed concurrently, increasing the real-life relevance of the exposures [27, 28]. By filtering particles out of the sample, exposure to the gaseous phase can be studied separately from the particulate effects. In vitro studies show that biomass combustion particles from several sources can cause cytotoxicity, inflammation and genotoxicity in several cell types [29–33]. We have reported similar responses in our previous in vivo studies [13, 34, 35]. Most previous toxicological findings on biomass combustion aerosols are from separately conducted in vitro and in vivo studies. These two study types usually provide complementary information on the toxicological mechanisms. However, in some cases, the findings contrast; for example, in our previous work [13, 14], biomass combustion emissions showed different in vivo and in vitro findings depending on the combustion efficiency and chemical composition of the emission particles. Simultaneous experiments reduce the risk of misinterpretation of the findings and can produce new insights into the toxicity of combustion-generated aerosols; thus, a more reliable technique to elucidate the toxicological effects and underlying mechanisms of emissions can be achieved by combining in vitro and in vivo methods and using novel toxicological methods. This approach can also be useful to evaluate which in vivo experiments could be replaced by in vitro experiments.

In this study, we used a murine in vivo inhalation protocol and three different ALI systems in concert with a comprehensive combustion emission characterization to investigate the toxicity of spruce and pine combustion emissions and suggest using these comprehensive settings in future aerosol research studies. First, we assessed the differences in the composition of spruce and pine combustion emissions with online and offline emission characterizations. Second, we investigated the differences in toxicological endpoints and systems biology approaches after exposure to murine macrophages (RAW264.7), human alveolar epithelial (A549) cells and mice. Third, we analysed the toxicity of the complete combustion emission (CCA) and high-efficiency particulate air (HEPA) filtered emission (HFA) on A549 cells. The exposures of the different models were as follows: A549 cells in Tox-ALI were exposed for 1 h per combustion condition, whereas the exposure duration for RAW 264.7 and A549 cells in OMICS-ALI was 4 h. All the cell exposures were performed in parallel with whole-body exposures of C57BL/6 J mice that were exposed to aerosol samples for 4 h in three consecutive days. Exposure durations were chosen according to previous results and pilot studies to achieve the highest value from each of the exposure systems. Exposures of A549 cells and mice were used to assess inflammatory markers, genotoxicity and cytotoxicity. RAW267.4 cells were used to compare systems biology approaches with the same effects in mice by the analysis and integration of transcriptome and proteome profiles. Additionally, to produce a comprehensive overview of the effects induced by the combustion aerosols, the chemical and physical properties of the combustion emissions were characterized by state-of-the-art mass spectrometry-based online and offline methods. Finally, a computer model approach was employed to determine both in vitro and regional lung PM deposition. This paper describes the characteristics of the combustion emissions and the toxicological responses in PM-exposed cell lines and mice with support of transcriptome and proteome analysis and discusses how aerosol composition affected the responses of cells and mice.

## Materials and methods

### Combustion emission characterization

#### Combustion and sampling setup

**Fig. 1:**
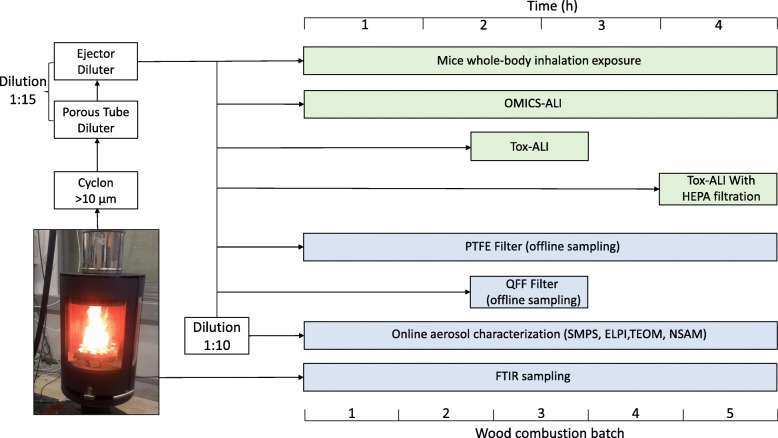
Schematic overview of in vivo and in vitro combustion emission exposure. Combustion aerosols emitted from the stove are led to a diluting sampling setup in which the final dilution ratio is set to 1:15 for toxicological exposures and 1:150 for online sampling of the aerosol composition. Diluted emission was directed into three different ALI systems, OMICS-ALI (two ALI systems) and Tox-ALI, as well as a mouse whole-body inhalation chamber, a gaseous online emission analyser (FTIR), and offline filter samplers. Exposure units are indicated in green and aerosol characterization processes in blue

Exposure experiments were conducted on three consecutive days each using wood logs of two common European softwood species, spruce (*Picea abies*) and pine (*Pinus sylvestris*), as fuel in a modern, non-heat-retaining chimney stove (Aduro, model 9.3, Denmark). This type of stove is commonly used in household heating in Central and Northern Europe. Each combustion experiment included 5 batches, each consisting of 2 kg of log wood. The first batch was ignited with a lighter from the top using 150 g of wood shavings and splints as kindling whereas in the later batches, the logs were placed on top of the remaining glowing charcoal. The combustion cycle was completed by emissions from the final glowing embers of the last batch for 25 min, leading to a total experimental duration of 4 h (Fig. 1).

A partial exhaust flow from the stack was guided through a pre-cut cyclone (> 10 μm) to remove possible unwanted stack-derived coarse particles from the sample stream. This was followed by two sample dilution steps consisting of a combination of a porous tube diluter and an ejector diluter (Venacontra, DAS, Finland). The combustion emission was diluted with purified compressed air (Aadco Inst., 737-series, USA) at room temperature using a dilution ratio of 1:15 to achieve the exposure emission used in the immediate in vitro and in vivo exposures. For online aerosol measurements, a diluted sample stream at 1:15 dilution was diluted further by a factor of 10 using an ejector diluter (Palas GmbH, VKL 10 E, Germany), leading to a total dilution ratio of 1:150 to reach suitable concentrations for the instruments.

#### Online aerosol measurements

A Fourier transform infrared analyser (FTIR; Gasmet Technologies Oy, model DX4000, Finland) was used for the continuous measurement of gaseous emissions directly from the undiluted exhaust gas, including CO_2_, NO_x_, CO, SO_2_, and several gaseous organic compounds typical for combustion processes. The sampling for FTIR was done using a heated sampling probe (Model PSP4000-H, Gasmet Technologies Oy). The size distribution and number concentration of the particulate emissions were measured using a scanning mobility particle sizer (SMPS; TSI Inc., model 3080, USA) to calculate the mass median (mobility) diameter (MMD) and an electrical low-pressure impactor (ELPI; Dekati Ltd., ELPI 10 lpm, Finland). In addition, the total suspended particulate mass (TSP) was measured with a tapered element oscillating microbalance (TEOM; Thermo Scientific, model 1405, USA). A nanoparticle surface area monitor (NSAM; TSI Inc., model 3550, USA) was used to measure the lung deposited surface area (LDSA) of the particulate matter, which corresponds to particle deposition efficiency in different compartments of the lungs.

#### Offline aerosol sample collection

For chemical analysis of emission contents, samples of the fine particulate matter (PM_1_, diameter < 1 μm) were collected onto quartz fibre (QFF) and Teflon (PTFE) filters for each whole 4 h exposure experiment (Fig. 1). Furthermore, QFF filters were collected during the 1 h unfiltered Tox-ALI exposure time.

#### Organic compound analysis

Organic and elemental carbon were measured using a thermal-optical carbon analyser (Magee Scientific, DRI Model 2001A, USA) following the Improve A protocol [36]. In situ derivatization and thermal desorption - gas chromatography - time-of-flight mass spectrometry (IDTD-GC-ToFMS, [37]) were used to analyse several of the organic target analytes (PAHs, oxygenated derivatives of PAHs (o-PAHs), anhydrosugars and resin acids) from QFF. Prior to the analysis, filter punches were prepared in GC liners. Ten microliters of methyl-trimethylsilyl-trifluoroacetamide (MSTFA, Macherey-Nagel) was added automatically to each liner by the sampling robot (Atas GL, PAL Focus, Netherlands) before the samples were placed in a direct thermal desorption unit (Atas GL, Linex and Optic 3, Netherlands) mounted to the gas chromatograph. During 16 min of thermal extraction, MSTFA was continuously added to the carrier gas stream at 4 mL min^− 1^. After thermal extraction and derivatization, the flow was set to pure carrier gas and reduced to 0.7 mL min^− 1^ with a split flow of 50 mL min^-1,^ and the GC-MS run was started using a BPX5, 25 m, 0.22 mm ID, 0.25 μm film thickness capillary column (SGE, Australia) installed in an Agilent 6890 gas chromatograph (Agilent, USA). Mass spectrometric detection in the range of 35 to 500 m/z was carried out on a Pegasus III ToFMS (Leco, USA) using an acquisition frequency of 25 spectra per second. The mass spectra were evaluated with the ChromaTOF software package (LECO, USA). Calibration and quantification were performed with mixtures of isotope-labelled internal standards and calibration standards (Supplementary Table 1, Additional file 1).

#### Elements

Elemental detection on the PTFE filters was performed by inductively coupled plasma atomic emission spectroscopy (ICP-AES; Perkin Elmer, Optima 7300 DV, Germany). Samples were transferred into closed quartz vessels and digested with HNO_3_ in a microwave system (Anton Paar, Multiwave 3000, Austria). The resulting solution was brought to a volume of 30 mL with ultrapure H_2_O. The following elements were determined from the emission samples: Al, As, B, Ba, Be, Bi, Ca, Cd, Co, Cr, Cu, Fe, Hg, K, Li, Mg, Mn, Mo, Na, Ni, P, Pb, S, Sb, Se, Sn, Sr, Ti, V, W, and Zn. Sample introduction was carried out by a peristaltic pump connected to a micromist nebulizer with a cyclone spray chamber. The radio frequency generator power was set to 1400 W, the plasma gas was argon with a flow rate of 15 L min^− 1^, and the nebulizer gas was argon with a flow rate of 0.6 Three blank determinations and a control determination of a certified standard (CPI) for all mentioned elements were performed regularly. Calculations were performed on the results on a computerized laboratory-data management system, relating the sample measurements to calibration curves, blank determinations and control standards.

### ALI exposure and cell culture

#### ALI systems used in exposure

We used three ALI systems optimized for different purposes in the in vitro studies, with two different deposition characteristics. A thermophoretic exposure system (Tox-ALI) [27] was used to assess cell death, DNA damage and cytokine release following 1 h exposures of A549 cells due to the rapid and high deposition of PM in this exposure system. Two automated exposure systems based on Vitrocell® technology (Vitrocell Systems, Germany) were used to expose cells that were later used in lactate dehydrogenase (LDH), transcriptome and proteome analyses. These two ALI systems, later referred to as OMICS-ALI, were used to expose A549 and RAW264.7 cells either with high-voltage PM deposition enhancement (1 kV; enhanced deposition) or without it (normal deposition) [38]. Before the aerosol was directed into each of the cell exposure units, the diluted (dilution ratio 1:15) aerosol sample was conditioned to attain optimal conditions for the exposed cells. For the Tox-ALI, the main flow of 5 L min^− 1^ was conditioned in a humidifier to approach 100% relative humidity while still staying below the condensation point at 37 °C in a partial flow of 150 mL min^− 1^, which led to the cell surfaces as a laminar flow. For OMICS-ALI systems, the diluted aerosol was conditioned to a relative humidity of 85% at + 37 °C and directed through the exposure units at a flow rate of 100 mL min^− 1^ [39]. Following Tox-ALI exposure, A549 cells were incubated for 24 h, while OMICS-ALI endpoints were measured directly after the exposure. In all ALI systems, complete combustion aerosol (CCA) emission (particle phase and gas phase) was used for exposures. In the Tox-ALI exposure, we used HEPA-filtered aerosols (HFAs) to study the effects of the gas phase. In addition, Tox-ALI exposures were performed in incubators with and without CO_2_, similarly to exposed cells. Clean air-exposed cells were used as controls in all experiments in OMICS-ALI. Positive controls were used as described below for specific endpoints. All experiments were performed in three independent exposures, which included several exposed wells and controls. The Tox-ALI exposure system systematically reduced viability in both the clean air controls and the exposure group. This did not hamper the comparison of the samples. The viability issue was tracked to the design the system and has been fixed in a later version.

#### Cell culturing for in vitro exposures

The cell lines A549, human alveolar basal epithelial cells, and RAW264.7, murine macrophages (ATCC®, CCL-158™ and ATCC®, TIB-71™), were exposed in ALI systems. Both cell lines were cultured in a humidified incubator at + 37 °C and 5% CO_2_ in either Dulbecco’s modified Eagle medium (DMEM) or Roswell Park Memorial Institute (RPMI) medium supplemented with 10% (v/v) foetal bovine serum (FBS), 2 mM L-glutamine, 100 U mL^− 1^ penicillin/streptomycin (Sigma-Aldrich or Gibco, Life Technologies). DMEM was used for cells exposed to the Tox-ALI system and RPMI medium for cells exposed to the OMICS-ALI system. Cells cultured for exposure to Tox-ALI were either seeded at a concentration of 250,000 cells 4 to 5 days prior to exposure or 180,000 cells 6 days prior to exposure on a 24 mm Falcon™ insert (Corning, #353090, USA), depending on the exposure day during the campaign. ALI conditions were imposed 48 h prior to exposure by removing the medium from the basolateral compartment of the insert and replacing the medium in the apical compartment with medium containing 5% FBS. Twenty-four hours before exposure, the culture medium was changed to serum-free medium to prevent further cell proliferation. Furthermore, immediately before the exposure, the medium on the apical side was replaced with serum-free medium with 25 mM 4-(2-hydroxyethyl)-1-piperazineethanesulfonic acid (HEPES) buffer (Sigma-Aldrich). Following Tox-ALI exposure, the culture medium was replaced with fresh serum-free medium. Cells were then incubated for 24 h, after which the culture media were collected and stored at − 80 °C for subsequent cytokine analyses. Cells were washed once with phosphate-buffered saline (PBS) and detached from the insert by trypsin-ethylenediaminetetraacetic acid (EDTA) treatment for 5 min at 37 °C. Thereafter, 100 μL of FBS was added to inhibit trypsin action, and the cells were rinsed using PBS with 10% FBS and centrifuged (500 g, 5 min, + 4 °C). A portion of the centrifuged cells were then resuspended in PBS with 10% FBS for use in cell viability analysis whereas PBS suspensions without serum were used in mitochondrial superoxide and reactive oxygen radical species measurements. The remaining cells were frozen and stored at − 80 °C for subsequent genotoxicity analysis.

In OMICS-ALI experiments, 24 h prior to exposure, ca. 500,000 cells were seeded on Transwell® inserts (Corning, #3450, USA). For cell exposure, the apical culture medium was removed, and serum-free medium supplemented with 10 mM HEPES was provided in the basolateral compartment during the exposures. Immediately after the exposures, medium from OMICS-ALI-exposed cells was used to quantify the release of LDH as an indicator of plasma membrane integrity. These cells were used in transcriptome and proteome analyses.

### In vivo inhalation exposure of mice

#### Animals and exposure

Animal exposure was conducted using 8- to 9-week-old male C57BL/6 J mice (average weight 26.88 ± 0.9 g). Each exposure group consisted of 6 mice. The mice were obtained from the Laboratory Animal Center of the University of Eastern Finland and were housed in plastic cages covered with reusable filter animal cage cover (Tecniplast INC., USA) on aspen wood chips, with access to water and maintenance diet ad libitum. The mice had a 12-h diurnal light cycle. The room temperature was 24.3 ± 0.7 °C and the humidity 36.8 ± 8.8%. The Animal Experiment Board in Finland (Regional State Administrative Agency of Southern Finland) approved all the experiments, which were carried out in accordance with EU Directive 2010/63/EU for animal experiments.

The mice were transferred to the inhalation exposure laboratory 24 h before the first exposure. Mouse exposures were conducted in a controlled inhalation chamber equipped with an automated monitoring system (TSE-Systems, DACO, Germany), where CO, CO_2_ and O_2_ concentrations, humidity, temperature and pressure were monitored to verify the correct exposure conditions. The concentrations of measured gases from the exposure chamber followed those measured with gaseous compound monitors from diluted exhaust gas or emission. All precise exposure measurements were performed as mentioned in the combustion emission characterization from online and offline measurements and are shown in Fig. 1. The mice were exposed 4 h per day on three consecutive days parallel to ALI exposures to achieve comparable conditions in all exposures. During the exposures, the mice were housed in two stainless steel cages (L: 265 x W:205 x H:140 mm, maximum 6 mice per cage) with access to water and food ad libitum. In the exposure chamber, the airflow was set to 20 L min^− 1^ and the relative humidity to 30%. Clean air in the chamber was obtained from an Aadco 737 zero air generator (Aadco Inst., model 737 zero, USA). The mice were exposed to diluted (dilution ratio 1:15) CCA. The untreated mice were constantly kept on the transfer station (Allentown Inc., Allentown Phantom, USA), isolated from contaminants by laminar air flow. After the first and second days of exposure, the mice were transferred to an animal transfer station, and after the third exposure, both the exposed and untreated mice were euthanized by terminal anaesthesia with intraperitoneal injection of 60 mg kg^− 1^ pentobarbital. Blood from cardiac puncture was collected into serum separation tubes (Terumo, Capiject® T-MG, USA). Mice were cannulated with polyethylene tubing, and lungs were perfused with 0.9% sterile saline, followed by the collection of bronchoalveolar lavage fluid (BALF) with two volumes of saline (30 mL kg^− 1^). BALF was collected with a syringe, and both aliquots were returned to and drawn from the lungs three times to maximize the efficiency of cell collection. Both portions of collected BALF were combined into one Eppendorf® LoBind microcentrifuge tube (Eppendorf, Germany).

#### BALF sample preparation

BALF cells were separated by centrifugation (500×g, 5 min), and the supernatant was collected and frozen at − 80 °C for cytokine analysis. The resulting cell pellets were resuspended in PBS containing 2% FBS (Sigma-Aldrich), and cell suspensions were used for total cell number count and cell differential measurements. The rest of the BALF was divided into two parts; freezing medium (50% RPMI, 40% FPS and 10% dimethyl sulfoxide (DMSO); all Sigma-Aldrich) was added to the BALF cells for analysis in the comet assay. RLT plus buffer (Qiagen) was added to the other part for omics analyses. Both BALF cell samples were frozen at − 80 °C for subsequent analyses.

#### In vivo single-cell suspension

A single-cell suspension of the mouse lung tissue was prepared according to the manufacturer’s instructions and methods described elsewhere [40]. The tissue was homogenized using enzymatic digestion, solutions A and D (Miltenyi Biotec), and mechanical dissociation steps using a lung dissociation buffer (Miltenyi Biotec) and a GentleMACS dissociator (Miltenyi Biotec, Germany). After homogenization, the samples were processed through a cell strainer (40 μm mesh size, Falcon™) to remove remaining larger lung tissue fragments. A single-cell suspension was measured for viability immediately after preparation and frozen at − 80 °C in a similar manner to the BALF samples for genotoxicity measurement.

### Estimation of the depositions

#### In vitro depositions

An estimation of the deposited particle mass, surface area or number can be derived from the measured exposure mass concentration, for instance, from PM data. In the first step, the particle-size-independent deposition rate is assumed for the OMICS-ALI systems, as suggested by several authors [41, 42]. The deposition function shows a relatively wide minimum in the nanoparticle size range between 100 nm and 600 nm. Aged particle populations usually show their concentration maximum at this size range. Assuming additionally that all the system-specific deposition-relevant parameters, such as flow patterns and gravitational or centrifugal forces, can be represented by one single overall particle deposition fraction *DF*, the deposition on the cell-covered surface area in the insert can be roughly estimated from:

1 $$ {TDD}_{ALI}=\frac{C_e\  DF\ {t}_e\ Q}{\pi\ {R}_{tw}^2} $$

with

*TDD*_*ALI*_ deposited mass per membrane insert area

*C*_*e*_ exposure concentration

*DF* deposition fraction

*t*_*e*_ experiment duration

*Q* flow rate for the exposure membrane insert

*R*_*tw*_ radius of the membrane insert.

For the OMICS-ALI system [41, 43], *DF* is estimated to be 1.5% [44], the exposure time is set to 4 h, the flow rate to 100 ml min^− 1^, and 2 *R*_*tw*_ to 24.4 mm, whereas for Tox-ALI, thermophoresis acts much more efficiently on particle deposition than diffusion and sedimentation [27] and has been reported to have a much higher deposition efficiency of 31%. The higher *DF* allowed a shorter exposure time of only 1 h. The flow rate was 150 ml min^− 1^, and the membrane insert diameter was 23.1 mm. The use of the electrostatic enhancement in the OMICS-ALI is resulting in a deposition enhancement by a factor of 3.9 for wood smoke [28].

Equation 1 is modified to eq. 2 to apply a similar estimation of deposited particle mass in the mouse lung. The breathing minute volume replaces the flow rate *Q,* which is derived from both the breathing tidal volume *V*_*t*_ and the breathing frequency *f*_*br*_. The size and number of lung pneumocytes [45] of the murine respiratory tract are used to estimate the dimensions of the alveolar epithelium and estimate the acinar or extra thoracic area *A*_*lung*_, where the particles are assumed to be deposited during inhalation:

2 $$ {TDD}_{lung}=\frac{C_e\ {DF}_{lung}\ {t}_e\ {V}_t{f}_{br}}{A_{lung}} $$

#### In vivo deposition

The breathing and deposition characteristics of [46] are used here as the body weight of their Balb/c mice of 25 g is close (− 7.2%) to the weight of our mouse strain (C57BL/6 J mice, body weight 26.9 ± 0.9 g). Accordingly, 0.2 mL and 300 min^− 1^ are taken here as the tidal volume and breathing frequency, respectively. The particle size-dependent acinar deposition function for mice [46] is applied to estimate the lung deposition fraction *DF*_*lung*_ at the modal diameter of the particle mass distribution. The tissue area of the alveolar epithelium for mouse species *A*_*lung*_ is reported as 500 cm^2^ by allometric analyses [45].

The size distribution of the wood combustion emission exposure aerosol was analysed and fitted by a lognormal mathematical function. The count median diameter (*CMD*) and geometric standard deviation (*σ*_*g*_) were found to be CMD = 91 nm & σ_g_ = 2.0 and CMD = 110 nm & σ_g_ = 1.8 for spruce and pine combustion, respectively. Conversion to MMAD resulted in 181 nm and 192 nm, respectively, while the size-dependent effective particle density for the wood combustion flaming phase [47] was used to transform CMD to CMAD and the Hatch-Choate-Conversion [48] for lognormal distributions to transform CMAD to MMAD.

Acinar deposition fractions of *DF*_*a*_ = 0.05 and 0.06 are derived from Fig. 5 from the study performed by Winkler-Heil and Hofmann [46] for spruce and pine, respectively; the deposition fractions are similar because the *DF*_*a*_ function is weakly size dependent beyond 200 nm. *TDD*_*al*_ is calculated from PM exposure concentration *C*_*e*_ using eq. 2 and the respective breathing data to provide results for *TDD*_*lung*_.

Deposited mass per tissue area (eqs. 1 and 2) is calculated for each mouse and ALI model to achieve a comparable data set. Additionally, the *TDD* per single cell is provided, as the type-I alveolar lung pneumocytes, which make up more than 90% of the lung epithelial cells, are known to be considerably larger in size than the A549 cells used for the ALI experiments. Assuming pneumocytes as the target cells builds in a roughly six-fold higher *TDD* if a cell count of 2.2·10^4^ cells cm^− 2^ is used for the mouse lung [45] and 1.3·10^5^ cells cm^− 2^ is used for ALI exposure [49]. Additionally, a life-span exposure TDD is calculated for the period between exposure start and experiment end to account for the three different “on-cell residence times” for a deposited particle, which are i) 1 h exposure and 23 h subsequent incubation for the thermo-ALI, ii) 4 h and no subsequent incubation for the mobile ALI, and iii) 4 h on each of three consecutive days for the mouse model. These residence times are chosen to mimic typical exposure scenarios such as short but intensive exposure followed by a recovery phase, a moderate but constant irritation, and daily exposure of a subject with overnight recovery.

### Transcriptome and proteome

#### Transcriptome sample collection and analysis

OMICS-ALI-exposed A549, RAW264.7, and mouse BALF cells were used to perform transcriptome analysis as described in detail in the supplementary material (Additional file 2). The methods used were previously reported by Oeder et al. [38]. Briefly, total RNA from A549, RAW264.7 and BALF cells was collected and amplified. The amplified cDNAs were hybridized, stained and scanned for transcriptome profiling. Transcriptome data were then analysed using Transcriptome Analysis Console (TAC; Thermo Fisher Scientific, version 4.0.0.25, USA).

#### Proteome sample collection and analysis

The proteome was analysed from OMICS-ALI-exposed RAW264.7 cells using methods previously reported by Sapcariu et al. [39]. A full description of the methods is given in the supplementary material, but briefly, the protein extracts of RAW264.7 cells were reduced and freed from sulfhydryl groups before the proteins were digested using sequencing grade endopeptidase LysC (Wako) and sequencing grade trypsin (Promega). Digested and purified peptides were lyophilized and reconstituted on tri-ethyl ammonium bicarbonate (TEAB) before diethyl labelling. Labelled peptides were separated, ionized and sprayed into the mass spectrometer (Thermo Fisher Scientific, Q-Exactive Plus, USA) for scanning. The recorded spectral data were analysed using the MaxQuant software package version 1.5.2.8.

### Toxicological measurements from in vivo and Tox-ALI experiments

#### Cell viability

Tox-ALI-exposed A549 cells, H_2_O_2_-exposed A549 positive controls and single-cell suspensions derived from mouse lungs were analysed using a fluorescence-based imager (ChemoMetec A/S, Nucleocounter NC-3000™, Denmark). For cell count and cell viability, the cells were stained according to the manufacturer’s instructions with a viability staining solution of acridine orange and 4′,6-diamidino-2-phenylindole (DAPI), which discriminates between living and dead cells. Analyses were performed using an A8 slide (ChemoMetec A/S).

#### BALF cell count and cell differential

The total cell number and viability of BALF cells were measured by light microscopy using a Bürker chamber and the trypan blue exclusion method. Cell differential counting slides were prepared using Cellspin II (Tharmac GmbH, Germany) by centrifugation at 1000 rpm for 10 min. Slides were fixed with May-Grünwald-Giemsa dye for microscopy (Zeiss, Axio Observer Z1, Germany) analyses, and at least 300 cells (63x) were calculated from each stained slide. The means of macrophages, lymphocytes and other white blood cells were calculated for reporting and statistical analyses.

#### DNA damage

DNA damage was analysed from a mouse lung single-cell suspension, BALF and Tox-ALI-exposed cells stored at − 80 °C using a slightly modified alkaline version of single-cell gel electrophoresis, or the comet assay, as previously described by Jalava et al. [50]. Briefly, the prepared comet assay samples were stained with ethidium bromide, and 100 cells per sample were analysed using Comet assay IV software (Instem, UK). The positive controls were prepared by exposing A549 cells to methyl methanesulfonate (MMS) and mice to diesel exhaust. The results are reported as the median of the percentage of DNA in the tail.

#### Cytokines

Cytokines were measured using two different methods. The V-PLEX proinflammatory panel 1 Mouse Kit (Meso Scale Diagnostics, Rockville, Maryland, USA) on a Sector™ 2400A Image Reader (Meso Scale Discovery (MSD), USA) was used for mouse cytokines from BALF and serum. The V-PLEX Proinflammatory Panel 1 includes 10 cytokines (IFNγ, IL-10, IL-12p70, IL-1β, IL-2, IL-4, IL-5, IL-6, TNFα and KC). Concentrations were determined with Discovery Workbench 2006® (3.0.18) software using the curve fit model. Cytokines measured from BALF were then normalized for standardization purposes using the total protein concentrations of the corresponding BALF samples [51–53]. IL-8 from aerosol or H_2_O_2_-exposed A549 cell culture medium at 24 h after exposure was measured with enzyme-linked immunosorbent assay (ELISA) kits (R&D Systems) in a plate reader (PerkinElmer, VICTOR3™ Multilabel Counter model 1420–051, USA). The concentrations of IL-8 were calculated by interpolation from the standard curve (Cubic Spline) using WorkOut2.0 software.

#### LDH cytotoxicity assay

The LDH test (Roche) was performed with medium samples from OMICS-ALI according to the manufacturer’s instructions. Cell culture medium kept at the same CO_2_ levels as exposed cells was used as a blank. Non-exposed control cells were lysed with 1% Triton X-100 for 20 min prior to the end of exposures, and the relative LDH content was used as the highest LDH release achievable; the absorbance values were set as 100% toxicity. Measurements were performed using a VICTOR3™ multilabel plate reader (model 1420–051, PerkinElmer, USA).

### Statistical methods

Differences between the samples were analysed using Welch one-way analysis of variance (ANOVA). The results from mouse and cell exposures showing a *p*-value> 0.05 for the homogeneity of variance and < 0.05 for the Welch F-test were used in multiple pairwise comparisons by Dunnett’s T3 post hoc test. Exceptions were the comet assay sample groups in which the homogeneity of variance was not met but, due to the robustness of the Welch ANOVA, a pairwise test was performed. The measured differences were regarded as statistically significant at *p* < 0.05. All the data were analysed using SPSS Statistics version 25 (IBM®, USA). Transcriptome and proteome samples were analysed by utilizing the statistical programming environment R. Downstream analyses were generated using QIAGEN’s Ingenuity Pathway Analysis (IPA®; QIAGEN, USA). The scaled mean from the pairwise comparisons [(mean difference/compared mean) × 100%] and 95% confidence interval (CI) were calculated [(95% CI upper or lower/compared mean) × 100%] and used to describe the percent difference between each pair of samples in the results.

## Results

### Combustion emission characteristics

#### Particle mass and number size distributions and gases in exposure emissions

**Fig. 2:**
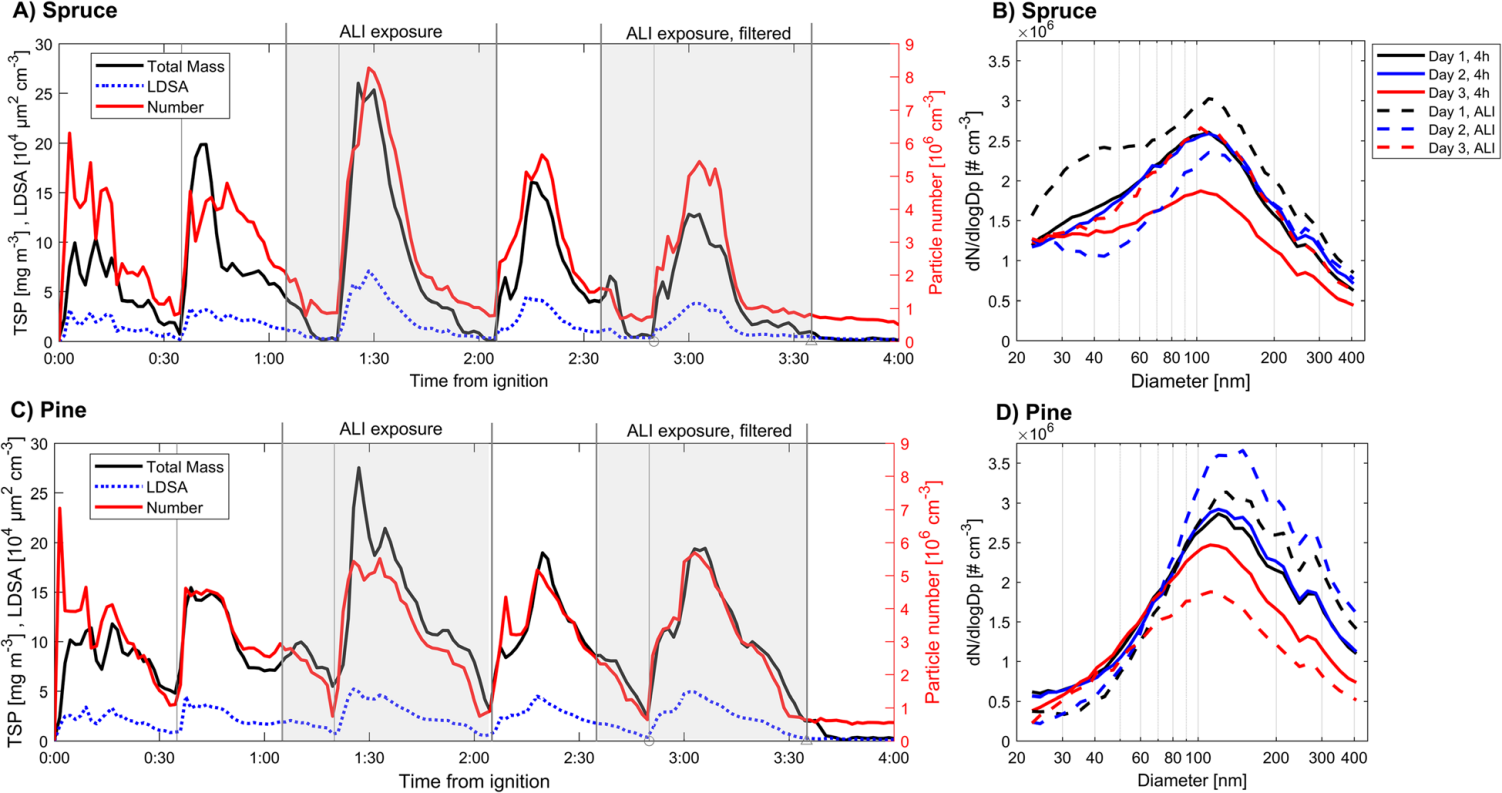
Physical properties of the diluted exposure exhaust for spruce **a-b** and pine **c-d** experiments. Parts A and C show the average over 3 experimental days of total suspended particulate mass (TSP; TEOM), lung deposited surface area (LDSA; NSAM) and particle number concentration (PNC, ELPI) during exposures, with Tox-ALI exposure times indicated with grey areas. Grey lines denote batch starting times, whereas size distribution during each exposure is shown in parts B and D

The aerosol profiles of the combustion emissions from both wood types in our combustion experiments differed in total mass, LDSA, MMD and particle number concentration (PNC) when compared by single batches or daily averages (Fig. 2, Supplementary Figs. 1, 2 and 3, and additional files 3, 4 and 5). Moreover, the average fine particulate matter (diameter below < 1 μm; PM_1_) mass emissions factors from the 4 h spruce and pine combustion experiments were 26 mg MJ^− 1^ and 41 mg MJ^− 1^, respectively, as measured by SMPS. Consequently, the exposure aerosol from pine combustion contained almost 1.5 times higher fine particulate matter PM_1_ mass for pine than spruce (Table 1). Furthermore, this paper focuses on PM_1_ mass as primary metric for PM mass whereas TSP is used as indicator of mass in all particle sizes. Tox-ALI exposures were performed daily during two separate periods, one with CCA and another with HFA (Figs. 1 and 2). The comparable batches (end of second to third batch of each combustion experiment) of spruce and pine combustion revealed that during this time, the pine combustion emissions contained higher PM_1_ mass (7.7 mg m^− 3^) than the respective spruce combustion aerosol (4.9 mg m^− 3^). HFA exposures were performed during the fourth to fifth batch, where the PM_1_ mass before HEPA filtration was 4.8 mg m^− 3^ for spruce and 6.4 mg m^− 3^ for pine emission (Fig. 2a, c). The average geometric mean diameter (GMD) of the pine combustion particles was slightly larger (110 nm) than that of spruce combustion particles (91 nm). Number size distributions (Fig. 2b and d) showed that spruce combustion produced a higher number of smaller (< 80 nm) particles than the corresponding pine combustion. Organic carbon content was similar with both wood combustion emissions except in spruce exposure by the Tox-ALI, where more organic carbon was measured, increasing the organic/elemental carbon ratio. Overall, pine combustion produced more elemental carbon except of carbon monoxide (CO), which was much more abundant in spruce combustion emission. Table 1 contains the concentrations in the exposure aerosol and approximate emission conversion factor (ECF), which allow the pollutant concentrations in the exposure aerosol to be converted into corresponding emission factors in relation to the fuel energy (MJ).

**Table 1:** Physical properties of the particles and concentrations of gaseous emissions in spruce and pine combustion aerosols following 4 h of exposure and Tox-ALI

		Spruce		Pine	
parameter	Unit	4 h average	Tox-ALI	4 h average	Tox-ALI
NO	ppm	1.9 ± 1.2	1.9 ± 1.2	1.7 ± 0.8	1.7 ± 0.7
NO_2_	ppm	0.2 ± 1.1	0.2 ± 0.5	0.2 ± 0.4	0.2 ± 0.7
THC	ppm	12.3 ± 28.2	16.1 ± 27.8	9.3 ± 18.8	9.4 ± 23.8
CO	ppm	122 ± 141	142 ± 149	84 ± 94	67 ± 88
CO_2_	ppm	4500 ± 1700	4600 ± 1900	4300 ± 1400	4300 ± 1300
PM_1_ mass (SMPS)	mg m^− 3^	4.3 ± 5.1	4.9 ± 6.0	6.2 ± 5.1	7.7 ± 6.5
TSP mass (TEOM)	mg m^− 3^	6.0 ± 7.8	7.4 ± 9.1	9.6 ± 7.6	12.3 ± 10.2
PNC (SMPS)	1 × 10^6^ cm^− 3^	2.1 ± 1.5	2.4 ± 1.8	2.1 ± 1.3	2.1 ± 1.5
PNC (ELPI)	1 × 10^6^ cm^− 3^	2.7 ± 3.8	3.2 ± 3.5	2.9 ± 2.2	3.1 ± 2.1
LDSA (NSAM)	1 × 10^6^ μm2 cm^− 3^	16.9 ± 18.1	20.5 ± 22.7	21.7 ± 15.6	24.8 ± 17.9
MMD (SMPS)	nm	304 ± 167	322 ± 168	365 ± 146	414 ± 102
GMD (SMPS)	nm	91 ± 29.2	94.3 ± 32.3	110 ± 32.7	120 ± 27.9
OC	μg m^− 3^	570 ± 160*	2100 ± 320	790 ± 340	850 ± 80*
EC	μg m^− 3^	4100 ± 1300*	4500 ± 1200	7900 ± 1500	6600 ± 3700*
OC/EC		0.14 ± 0.01	0.47 ± 0.05	0.10 ± 0.03	0.20 ± 0.12*
ECF	m3 of exposure aerosol /MJ fuel energy	11.0 ± 1.2	11.0 ± 1.4	11.3 ± 0.2	11.4 ± 0.3

*THC* total hydrocarbon, *PM*_*1*_ particulate matter < 1 μm, *TSP* total suspended particles, *PNC* particle number concentration, *LDSA* lung deposited surface area, *MMD* mass median mobility diameter, *GMD* geometric mean mobility diameter, *OC* organic carbon, *EC* element carbon, *ECF* approximate emission conversion factor.

The values shown are calculated as averages from three test cycles ± standard deviation. Note that standard deviations have been calculated on the online data. Approximate emission conversion factor (mean of experiment-wise averages ± standard error of the mean) enables conversion to correspond emission factor. *Denotes only 2 days of measurements

#### Organic compounds in the emissions

Pine emission particles generally showed two- to three-fold higher concentrations of PAHs and o-PAHs than the spruce combustion particles (Table 2). In addition, the distribution of particle-bound organic components from pine combustion was rather similar to that of spruce combustion. A more comprehensive list of measured organic compounds is shown in Supplementary Table 2 and additional file 6. The combustion emissions used in exposures showed high concentrations of well-known hazardous compounds, such as the carcinogenic PAH benzo [a] pyrene, which was three-fold higher in pine emission (6.6 μg m^− 3^) than in spruce emission (2.1 μg m^− 3^). Most toxic PAH compounds were determined using the toxic equivalency factor (TEF; DRG [97]) to be dibenzo [al] pyrene with a toxic equivalent (TEQ) value of 27.

**Table 2:** Organic compounds from wood combustion emissions. Organic compounds were sampled over 4-h exposure. Concentrations are calculated as averages from three test cycles ± standard deviation

Parameter	TEF	spruce (μg m-^3^)	TEQ	pine (μg m-^3^)	TEQ
**PAH**					
Benzo [a]pyrene	1	2.10 ± 0.79	2.1	6.6 ± 3.4	6.6
Dibenz [ah]anthracene	1	0.13 ± 0.039	0.13	0.42 ± 0.043	0.42
Dibenzo [al]pyrene	10	1 ± 0.48	10	2.7 ± 1.4	27
Dibenzo [ae]pyrene	1	0.33 ± 0.21	0.33	0.76 ± 0.67	0.76
Dibenzo [ai]pyrene	10	0.13 ± 0.06	1.3	0.36 ± 0.37	3.6
Dibenzo [ah]pyrene	10	0.091 ± 0.051	0.91	0.2 ± 0.21	2
**alkyl-PAH**					
9-Methylphenanthrene		0.29 ± 0.42		0.41 ± 0.58	
3,6-Dimethylphenanthrene		0.005 ± 0.008		0.006 ± 0.009	
Retene		0.26 ± 0.21		0.61 ± 0.03	
7,12-Dimethyl-Benzo [a]anthracene		0.14 ± 0.075		0.21 ± 0.3	
1-Methylbenzo [a]anthracene		0.018 ± 0.011		0.033 ± 0.047	
**o-PAH**					
9H-Fluoren-9-one		12 ± 6.6		38 ± 27	
1H-Phenalen-1-one		85 ± 22		180 ± 130	
**Carbonyls**					
formaldehyde		377 ± 49		311 ± 28	
acetaldehyde		206 ± 64		152 ± 21	
acrolein		16.6 ± 1.2		14.9 ± 0.7	
**Anhydrosugars**					
Galactose		0.095 ± 0.04		0.35 ± 0.15	
Mannose		0.28 ± 0.053		0.62 ± 0.13	
Levoglucose		3.5 ± 1.9		4.9 ± 3.6	
**Resin acids**					
Isopimaric acid		0.005 ± 0.006		0.003 ± 0.002	
Dehydroabietic acid, methyl ester		0.029 ± 0.029		0.049 ± 0.007	
Dehydroabietic acid		1.1 ± 1		1.4 ± 0.25	
Abietic acid		0.012 ± 0.004		0.024 ± 0.006	

*TEF* Toxic Equivalency Factor, *TEQ* Toxic Equivalents.

#### Inorganic elements in PM emissions

The three most abundant elements in combustion emissions were K, S and Zn, with concentrations in spruce combustion emissions of 124.0, 17.0, and 11.9 μg m^−3^ and in pine combustion emissions of 75.1, 8.6 and 6.9 μg m^− 3^, respectively. Notable deviations in concentration between wood types were observed for Sn, Ba, Cu, Fe, W, S, Na, Sr, Pb, Zn, Li, K and Mg, with spruce combustion showing generally higher concentrations of all elements except Mg, which was detected in higher concentrations in pine combustion emissions (see Supplementary Table 3, Additional File 7).

### Toxicological effects in vitro and in vivo

#### Estimation of deposited PM

The deposition of PM in mouse lungs in whole-body inhalation exposure and in both ALI systems revealed expected differences in deposited PM mass, concentration and LDSA (Table 3). The highest deposition was achieved in OMICS-ALI with enhanced deposition at 4 h exposure to pine combustion emission. The normal deposition in OMICS-ALI and Tox-ALI showed rather similar depositions over the exposure duration, even though the Tox-ALI exposure lasted a quarter as long as the OMICS-ALI exposure. In mouse lungs, the aerosols were estimated to deposit at a much lower amount (32.1 ng cm^− 2^), and the mice were exposed for three consecutive days. High standard deviation (SD) values in deposition estimations are mostly caused by variation in the PM_1_ measurements during the 4 h batch combustion exposures, and such variation may reduce the reproducibility of cell exposures.

**Table 3:** Estimations of deposited concentrations of PM_1_, TSP and LDSA in the in vivo, OMICS-ALI and Tox-ALI exposures

			Spruce				Pine			
parameter	Unit	device	In vivo	OMICS-ALI		Tox-ALI	In vivo	OMICS-ALI		Tox-ALI
				Normal deposition	Enhanced deposition			Normal deposition	Enhanced deposition	
PM_1_	ng cm^−2^	ELPI	18.6 ± 22.0	380 ± 462	1480 ± 1800	470 ± 95.3	32.1 ± 26.4	590 ± 500	2300 ± 1950	690 ± 360
TSP	ng cm^− 2^	TEOM	25.9 ± 34	570 ± 701	2220 ± 2730	660 ± 180	50.0 ± 39.0	950 ± 790	3710 ± 3080	1100 ± 640
LDSA	1x10^6^μm^2^cm^− 3^	NSAM	73.0 ± 78	1.6 ± 1.7	6.2± 6.6	1.84 ± 0.41	110 ± 81.0	1.91 ± 1.38	7.4 ± 5.4	2.23 ± 0.01

*PM*_*1*_ Particulate matter with aerodynamic diameter < 1 μm, *TSP* total suspended particles, *LDSA* lung deposited surface area.

The values shown are calculated as averages from three test cycles ± standard deviations

#### In vitro toxicity

**Fig. 3:**
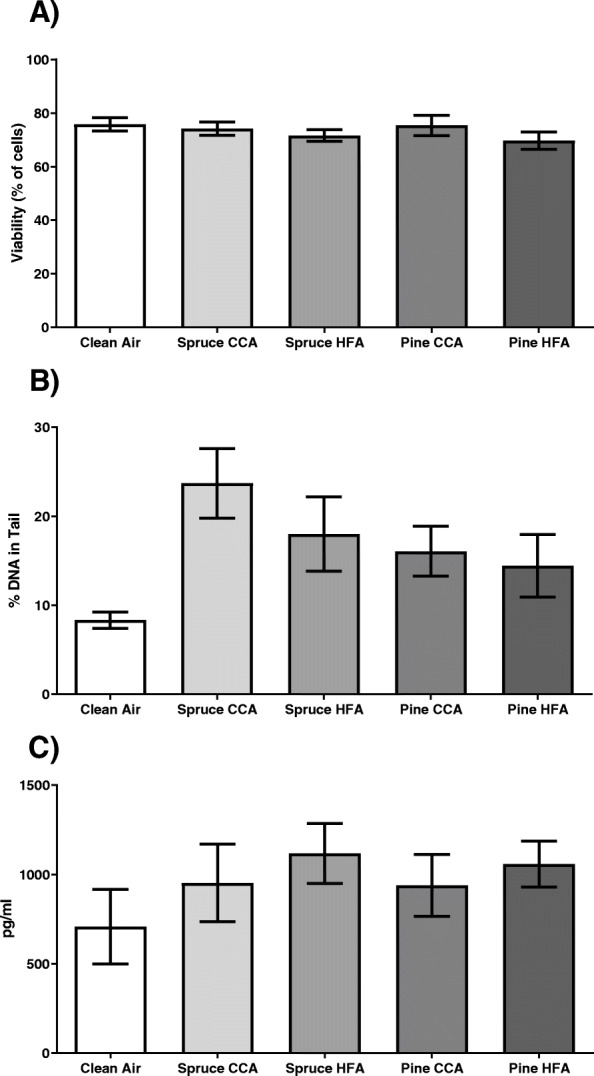
Viability **a**, comet assay **b** and IL-8 secretion **c** of A549 cells after exposure for 1 h at Tox-ALI to spruce, pine or clean air. Each bar shows the mean ± SEM, *n* = 3 for cell exposure. CCA = complete combustion aerosol, HFA = HEPA-filtered aerosol-exposed cells

In the Tox-ALI exposed cells, the different emissions showed no impact on the viability of the cells measured with viability staining (Fig. 3a). The clean air control, however, showed nonsignificant decreased viability compared to the incubator controls with or without CO_2_ (Supplementary Fig. 4A, additional file 8). This decreasing effect was a result of a systematic reduction in viability due to the Tox-ALI exposure system, which affected all the exposed cells similarly and therefore allowed sample comparison.

Both the wood fuel CCA- and HFA-exposed cells revealed a nonsignificant increase in DNA strand breaks compared to the clean air control (Fig. 3b). DNA damage was strongly induced by spruce CCA emission, which increased the amount of damaged DNA by 166% (95% CI: − 10.193, 342% *p* = 0.058) compared to the clean air control. The HFA-exposed samples were 24% (95% CI: − 43.141, 91.348%) lower than the CCA-exposed samples. Between the wood fuel types, the amount of DNA damage in pine combustion aerosol-exposed cells was 47% (95% CI: − 40.541, − 135.820%) lower than that in spruce-exposed cells (CCA exposures). Furthermore, there were no differences between pine CCA and HFA exposure in the DNA damage measurements. However, the incubator controls showed lower amounts of damage, especially the incubator controls without CO_2_ (Supplementary Fig. 4B, additional file 8)_._

Inflammatory responses of A549 cells were detected by measuring IL-8 levels from cell culture medium at 24 h post-exposure incubation (Fig. 3c). A slight trend, although nonsignificant increases in IL-8 levels were observed following all exposures, especially in HFA-exposed samples. HFA-exposed samples showed slightly higher inflammatory responses than the corresponding CCA-exposed samples for both wood types. Furthermore, clean air-exposed cells produced 707 pg mL^− 1^ IL-8 in the cell culture medium, which was similar to the CO_2_-deprived incubator control sample, i.e., 689 pg mL-1 IL-8, under the same conditions without air flow (Supplementary Fig. 4C, additional file 8).

#### Cytotoxicity of cells at OMICS-ALI

**Fig. 4:**
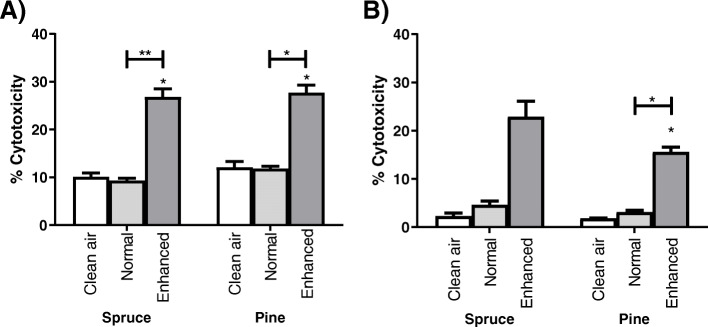
Cytotoxicity measured via LDH release of OMICS-ALI-exposed RAW264.7 **a** and A549 cell lines **b** following exposure to the combustion emission of spruce, the combustion emission of pine, or clean air. * Denotes a significant difference (*p* < 0.05) from clean air-exposed cells, CA clean air control, AS aerosol deposition, HV enhanced deposition with high voltage. Each bar shows the mean ± SEM, n = 3

To avoid severe cytotoxicity in OMICS-ALI samples, LDH release was measured in RAW264.7 and A549 cells following exposure. LDH release showed significantly elevated levels of cytotoxicity compared to the controls only after enhanced deposition for both wood combustion emissions but was still being in a range that allowed use for the OMICS analysis (Fig. 4).

The RAW264.7 cells showed no cytotoxic response after exposure to the spruce combustion emission when compared to the corresponding clean air exposure (Fig. 4a). Cytotoxicity became significant after exposure to spruce CCA was 165% (95% CL: 115, 214%, *p* = 0.002) higher and pine CCA 78% (95% CL: 56.3, 201%, *p* = 0.007) higher than the clean air controls when enhanced deposition was used. In addition, enhanced deposition exposure displayed substantially higher cytotoxicity than non-enhanced deposited spruce CCA by 2.8-fold (95% CI in folds: 2.28, 3.44, *p* = 0.003) and pine CCA by 1.7-fold (95% CL in folds: 1.51, 3.20, *p* = 0.017).

Similarly, in the A549 exposures, deposited emissions barely increased cytotoxicity after exposure to both CCA samples compared to the clean air-exposed cells (Fig. 4b). Only after enhanced deposition exposure to pine was CCA cytotoxicity significantly increased, being 7.6-fold (95% CI in folds: 4.64, 12.70, *p* = 0.014) higher than clean air and 4-fold (95% CI in folds: 3.03, 6.90, *p* = 0.008) higher compared to non-enhanced deposition exposure. Furthermore, even higher cytotoxicity up to 8.8-fold (95% CI in folds: − 2.49, 20.1, *p* = 0.068) was observed for spruce CCA after enhanced deposition exposure compared to clean air; however, this difference was not statistically significant.

#### In vivo toxicity

**Fig. 5:**
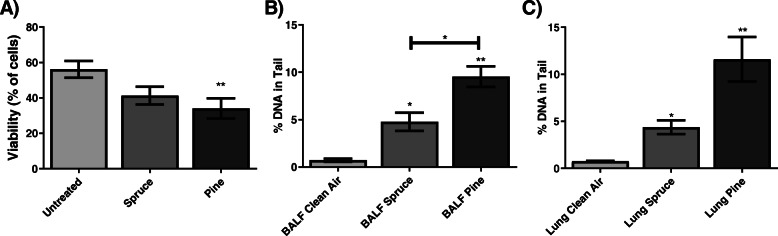
Viability of the lung cells in single-cell suspension **a**. DNA damage in the BALF cells **b** and the pulmonary cells of the lung tissue **c** after 3 exposures (4 h each) of healthy C57BL/6 J mice to combustion emission of spruce, pine or corresponding untreated mice (mean values ± SEM). A single asterisk indicates a statistically significant difference compared to untreated mice (*p < 0.05, **p < 0.01), *n* = 6

Generally, low viability was observed in all lung tissue homogenate cell samples in all tested groups, whereas BALF cells showed good viability for all test groups (Fig. 5a and Table 4). Viabilities measured from the lung single-cell suspension after exposure were 26% lower (95% CI: − 0.243, 75.670%, *p* = 0.137) for spruce CCA and 39% lower (95% CI: − 0.059, 75.670%, *p* = 0.033) for pine CCA than those from corresponding untreated mice. Both BALF cells and lung single-cell suspensions showed significantly increased genotoxic effects upon exposure compared to untreated samples (Fig. 5b, c). In the BALF samples, spruce CCA exposure induced over 10-fold higher (95% CI in fold-changes: 3.36, 18.00, p = 0.017) and pine CCA exposure over 21-fold higher (95% CI in fold-changes: 13.10, 29.70, *p* = 0.001) amounts of cells with DNA damage compared to the BALF of untreated mice. In addition, pine CCA exposure caused two-fold higher (95% CI in fold-changes: 1.14, 2.86, *p* = 0.024) DNA damage than spruce CCA in the BALF of exposed mice. In the lung single cell-suspension, spruce CCA exposure induced almost a 9-fold higher (95% CI in fold-changes: 3.84, 13.70, *P* = 0.008) and pine CCA exposure induced a 23-fold higher (95% CI in fold-changes: 7.13, 39.40, p = 0.014) amount of DNA damage than was detected in lung cells of untreated mice. Between wood types, only a small difference was observed, with pine emission inducing a 2.7-fold higher (95% CI in fold-changes: − 1.16, 4.47, *p* = 0.071) amount of DNA damage than spruce emission.

**Table 4:** Number of cells and cytokines from C57BL/6 J mice (*n* = 6) in the BALF and serum

Parameter	Unit	BALF			Serum		
		Untreated	Spruce	Pine	Untreated	Spruce	Pine
Total cell number	10^4^ cells mL^− 1^	50 ± 5.2	42 ± 7.1	117.6 ± 48.8	n.a.	n.a.	n.a.
Viability	%	82.5 ± 3.6	89.3 ± 3.4	92 ± 2	n.a.	n.a.	n.a.
Macrophages	10^4^ cells mL^−1^	49.4 ± 5.2	41.3 ± 7.1	117.1 ± 48.7	n.a.	n.a.	n.a.
Neutrophils	10^4^ cells mL^−1^	0.05 ± 0	0.3 ± 0.01	0.04 ± 0	n.a.	n.a.	n.a.
Lymphocytes	10^4^ cells mL^−1^	0.4 ± 0.2	0.4 ± 0.01	0.4 ± 0.2	n.a.	n.a.	n.a.
Total protein	μg mL^−1^	94.3 ± 5.2	67.4 ± 2.4	85.3 ± 10.5	n.a.	n.a.	n.a.
IFN-γ	Norm. pg mL^−1^	0.30 ± 0.06	0.45 ± 0.12	0.43 ± 0.17	1 ± 0.24	0.7 ± 0.07	3.2 ± 2.69
IL-10	Norm. pg mL^− 1^	2.37 ± 0.29	0.97 ± 0.47	3.05 ± 0.51	18 ± 0.97	**12 ± 1.16**	14 ± 1.99
IL-12p70	Norm. pg mL^− 1^	15.30 ± 9.64	20.05 ± 4.74	17.12 ± 5.60	15 ± 4.26	21 ± 6.16	21 ± 5.6
IL-1b	Norm. pg mL^−1^	0.85 ± 0.18	1.39 ± 0.12	0.88 ± 0.14	2 ± 0.15	2 ± 0.1	2 ± 0.1
IL-2	Norm. pg mL^− 1^	2.15 ± 0.46	3.53 ± 0.45	1.88 ± 0.50	2.4 ± 0.32	1.6 ± 0.21	1.7 ± 0.14
IL-4	Norm. pg mL^− 1^	0.93 ± 0.33	2.03* ± 0.16	1.20* ± 0.20	0.4 ± 0.06	0.8 ± 0.32	0.4 ± 0.16
IL-5	Norm. pg mL^−1^	1.68 ± 0.55	2.15* ± 0.36	0.86* ± 0.11	3.5 ± 1.48	2.2 ± 0.51	1.2 ± 0.12
IL-6	Norm. pg mL^− 1^	8.22 ± 1.68	16.44* ± 0.50	7.87* ± 1.52	6.2 ± 0.8	5.1 ± 0.52	5.3 ± 0.42
TNFα	Norm. pg mL^− 1^	4.30 ± 0.55	**6.58 ± 0.42**	5.03* ± 0.51	15 ± 0.96	11 ± 1.1	**9.3 ± 0.94**
KC	Norm. pg mL^−1^	14.74 ± 2.10	55.79* ± 7.55	28.46* ± 4.21	64 ± 4.18	56 ± 3.12	59 ± 3.44

Values are presented as the mean value ± SEM. Bold-faced number indicates a statistically significant difference from untreated mice (*p* < 0.05), * indicates a statistically significant difference between CCA exposures. Norm. pg mL-1 indicates standardization to the corresponding total protein amount; n.a. indicates not applicable

Combustion emission-induced inflammatory responses were studied in mice by detecting the number of inflammatory cells in BALF as well as by measuring the concentrations of ten different cytokines from BALF and serum samples (Table 4). The spruce CCA-exposed mice showed a similar total cell number in BALF as untreated mice, whereas pine combustion aerosol exposure increased the total cell number more than two-fold (95% CI in fold-changes: − 2.95, 5.60, *p* = 0.490) compared to untreated mice. A similar effect was also observed in macrophage numbers. A high variability in the cell numbers of pine CCA-exposed mice explains this observation since two of the six mice showed very large increases in BALF cell number. BALF of the spruce CCA-exposed mice showed a 5.5-fold increase (95% CI in fold-changes: − 1.73, 7.35, *p* = 0.089) in neutrophil numbers compared to untreated mice. Lymphocytes were observed only in very low numbers, and no changes were observed between the exposed and untreated mice.

Overall, the cytokine levels in BALF and serum after exposure were very low (Table 4). Statistically significant increases in BALF cytokine levels were observed for IL-4, IL-6, TNFα and KC after spruce CCA exposure compared to untreated mouse samples. IL-4 showed 84% (95% CI: − 0.002, 108, *p* = 0.05), IL-6 showed two-fold (95% CI in fold-changes: 1.03, 2.68 *p* = 0.010), TNFα showed 50% (95% CI: 5.370, 96.270%, *p* = 0.029) and KC almost four-fold (95%CI in fold-changes: 2.07, 5.50, *p* = 0.006) increases after exposure to spruce CCA when compared to untreated mice. Significant differences between the groups exposed to spruce and pine CCA were also observed. In the BALF of exposed mice, spruce CCA showed a 70% increase in IL-4 (95% CI: − 7.529, 131%, *p* = 0.028) and two-fold increases in IL-6 (95% CI in fold-changes: 1.44, 2.73, *p* = 0.005) and KC (95% CI in fold-changes: 1.05, 2.86, *p* = 0.038) concentrations compared to pine CCA-exposed mice. Moreover, a 2.5-fold increase in spruce CCA-exposed compared to untreated mice was observed for IL-5 (95% CI in fold-changes: 1.10, 3.89, *p* = 0.037). In contrast, in serum, after spruce CCA exposure, the levels of IL-10 (95% CI: − 0.006, 13.742%, *p* = 0.003) were 37% lower than those in untreated and pine CCA-exposed mice. Correspondingly, the TNFα (95% CI: − 0.019, 10.112%, *p* = 0.008) level was 36% lower than that in untreated mice.

### Transcriptome and proteome analysis

#### Transcriptome analysis

**Fig. 6:**
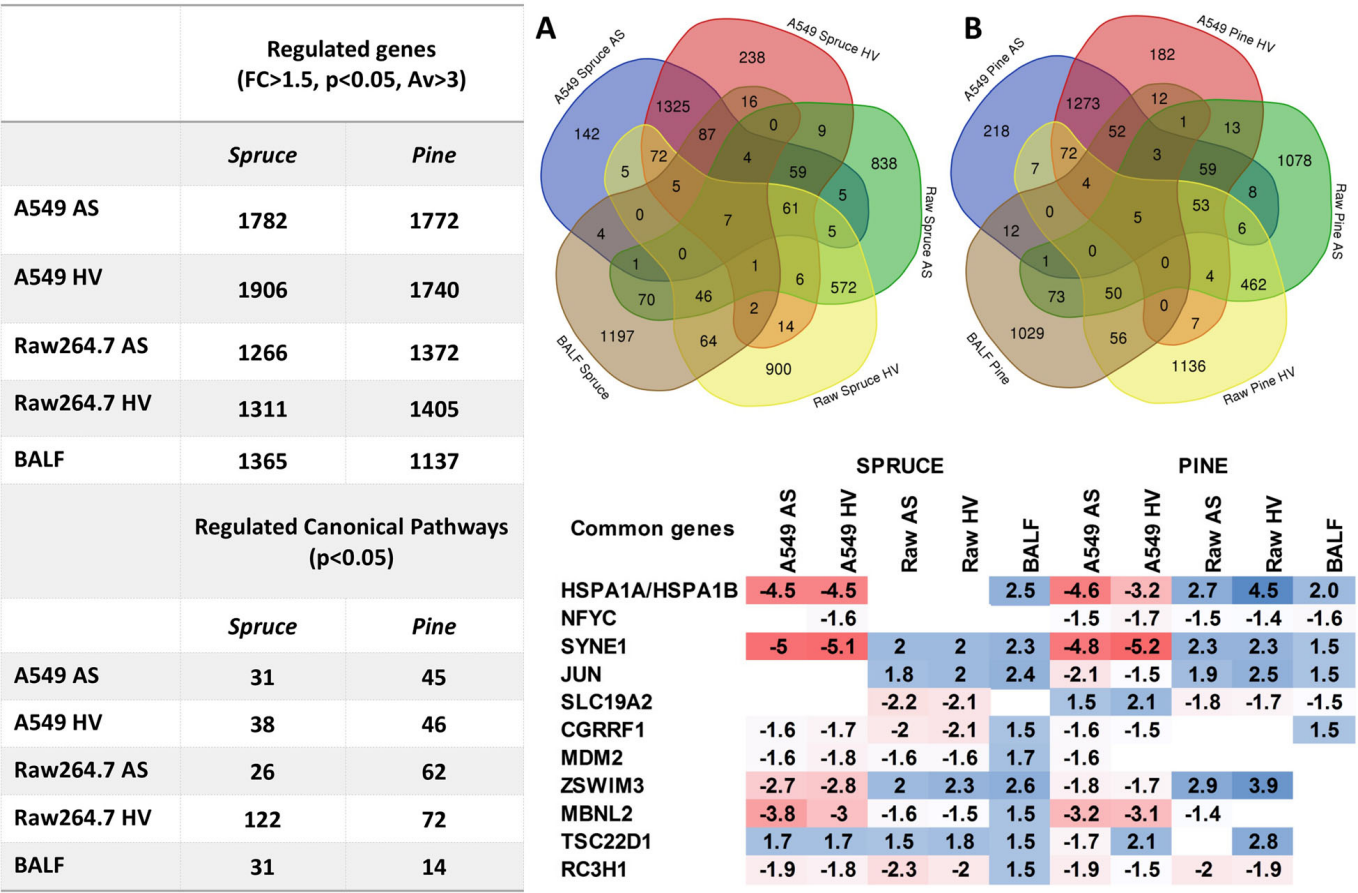
The numbers of significantly regulated genes and canonical pathways are shown in the tables. Venn diagrams of shared regulated genes following spruce **a** and pine **b** CCA exposures. The heat map shows fold-change values for the commonly regulated genes. AS aerosol deposition, HV enhanced deposition with high voltage

A variable number of genes and canonical pathways were regulated among the different cellular systems and conditions both in vitro and in vivo (Fig. 6). The Venn diagrams show the overlapping regulated genes following spruce (Fig. 6a) and pine (Fig. 6b) CCA exposure. The *HSPA1A* and *HSPA1B* genes, encoding stress-inducible heat shock proteins (Hsp70), were significantly downregulated in A549 cells and upregulated in both RAW264.7 cells and BALF by pine CCA exposure and to a lesser extent by spruce CCA exposure. The DNA binding nuclear transcription factor gamma (*NFYC*) was significantly downregulated by pine CCA exposure, while the thiamine transporter gene (*SLC19A2*) was upregulated in A549 cells and downregulated more effectively in both RAW264.7 cells and BALF following pine CCA exposure. The cell growth regulator *CGRRF1* was significantly downregulated in vitro and upregulated in vivo*,* particularly following spruce CCA exposure, as was the case for *MDM2* transcripts (murine double minute 2), a negative regulator of the tumour suppressor *p53*. In addition, the tumour suppressor gene *TSC22D1* was significantly upregulated both in vitro and in vivo*,* mainly following spruce CCA exposure. Interestingly, the *SYNE1* gene (spectrin repeat containing nuclear envelope protein 1) was upregulated in A549 cells and downregulated in both macrophages and BALF cells after exposure to both wood CCAs. The *SYNE1* gene was recently associated with the pathogenesis of asthma [54].

#### Canonical pathway analysis

Functional characterization of the differentially expressed genes, performed using IPA software, identified a variable number of regulated canonical pathways (Fig. 6). The top five significantly regulated pathways affected by spruce and pine CCA exposure are shown in Supplementary Tables 4 and 5 (additional files 9 and 10), respectively. Spruce CCA exposure induced acute in vivo inflammatory responses (TREM1 signalling) as well as alkaloid and polyphenol compound metabolic pathways. Glutathione detoxification mechanisms were also activated by in vivo spruce CCA exposure, as shown by a significant positive Z-score. Following pine CCA exposure, the most significant pathways regulated in vivo refer to immune responses, either in terms of the coordination of agranulocyte infiltration in inflammation sites or in terms of cross talk between the innate and adaptive immune systems. In addition, circadian rhythm signalling was also affected by pine CCA exposure in vivo.

Several canonical pathways were variously activated in vitro and in vivo*,* revealing significant differences between measured sample-specific toxicogenomic mechanisms (Table 5). *Glucocorticoid receptor signalling* and *NRF-2-mediated oxidative stress* pathways were significantly regulated both in vitro and in vivo following spruce CCA exposure. *Unfolded protein response* is the most commonly regulated pathway by both CCA exposures in vitro*,* while *aryl hydrocarbon receptor* signalling is more significantly activated following pine CCA exposure, as expected given the higher PAH content in pine emissions. *PPAR* signalling is significantly regulated following all in vitro pine CCA exposures as well as following high voltage increased deposition in spruce CCA exposures.

**Table 5:** Comparison of common regulated canonical pathways in vitro and in vivo as analysed by IPA software

Canonical Pathways	Spruce					Pine				
[−log(*p*-value)]	A549		RAW264.7		BALF	A549		RAW264.7		BALF
	Normal	Enhanced	Normal	Enhanced		Normal	Enhanced	Normal	Enhanced	
Glucocorticoid receptor signaling	1.28	1.60	0.58	1.13	2.19	2.42	2.31	2.12	2.37	2.79
Role of Macrophages, Fibroblasts and Endothelial Cells in Rheumatoid Arthritis	1.85	1.85	0.31	2.79	1.04	1.62	2.60	1.81	4.62	–
NRF2-mediated Oxidative Stress Response	1.49	1.90	1.82	1.26	1.55	2.23	1.54	3.57	2.19	0.62
TNFR2 Signaling	1.55	0.98	1.19	4.64	0.30	1.54	1.08	1.75	3.83	1.00
Aryl Hydrocarbon Receptor Signaling	1.03	1.82	1.45	2.23	1.10	3.13	3.21	0.85	2.30	0.69
IL-17A Signaling in Fibroblast	1.20	1.52	2.30	3.57	0.28	1.19	1.68	1.64	3.62	0.36
Role of PKR in Interferon Induction and Antiviral Response	2.84	3.22	–	2.24	–	2.82	2.30	–	2.92	–
IL-17A Signaling in Gastric Cells	0.84	1.20	3.20	3.90	0.40	0.83	1.32	0.99	2.25	1.24
p38 MAPK Signaling	2.04	1.77	0.96	1.39	0.61	3.10	2.42	1.23	1.79	0.23
p53 Signaling	1.15	1.49	2.08	2.64	1.01	1.70	1.45	1.65	1.55	0.24
GADD45 Signaling	0.70	1.10	2.66	3.39		1.18	1.20	1.83	2.54	0.54
Il-6 Signaling	0.68	1.11	1.15	2.63	0.74	1.06	0.89	2.50	3.59	0.38
Aldosterone Signaling in Epithelial Cells	1.03	1.96	0.50	1.44	1.09	2.90	2.66	1.66	0.57	0.78
IL-10 Signaling	0.95	0.83	1.73	1.55	0.35	1.25	0.97	2.71	3.70	0.48
Unfolded Protein Response	1.47	1.70	1.68	1.51	0.88	1.45	1.91	1.20	2.03	0.20
PPAR Signaling	1.24	1.63	0.92	1.38	0.42	1.84	1.56	2.38	2.21	0.29

#### Proteome analysis

**Fig. 7:**
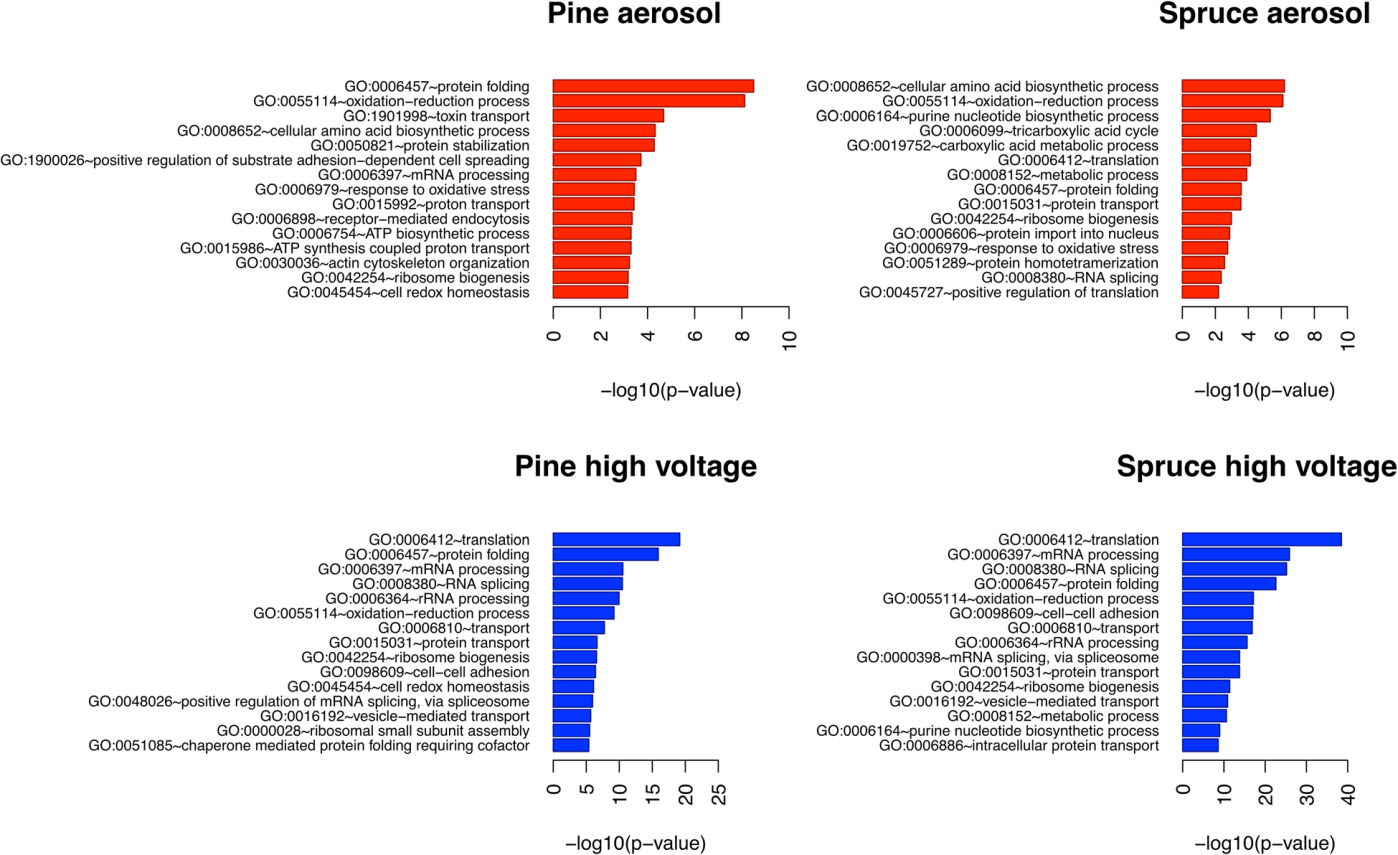
Top 15 regulated Gene Ontology (GO) biological processes in RAW264.7 cells at the proteome level after exposure to pine and spruce CCA (red) and to pine and spruce CCA with enhanced deposition at high voltage on ALI (blue)

The top 15 significantly regulated biological processes at the protein level are summarized in Fig. 7. Both spruce and pine aerosol exposure activated oxidation-reduction processes and oxidative stress responses. Moreover, pine combustion aerosol-exposed RAW264.7 cells showed induction of endocytosis. In response to spruce and pine CCA exposure and enhanced deposition, RNA- and transport-related processes were regulated. Following exposure to pine CCA with both normal and enhanced deposition, RAW264.7 cells tried to maintain homeostasis to compensate for the cytotoxic aerosol effect. In summary, exposure to spruce and pine CCA induced similar top 15 significantly regulated biological processes at the proteome level.

#### Comparison of the biological effects by proteome and transcriptome integration

**Fig. 8:**
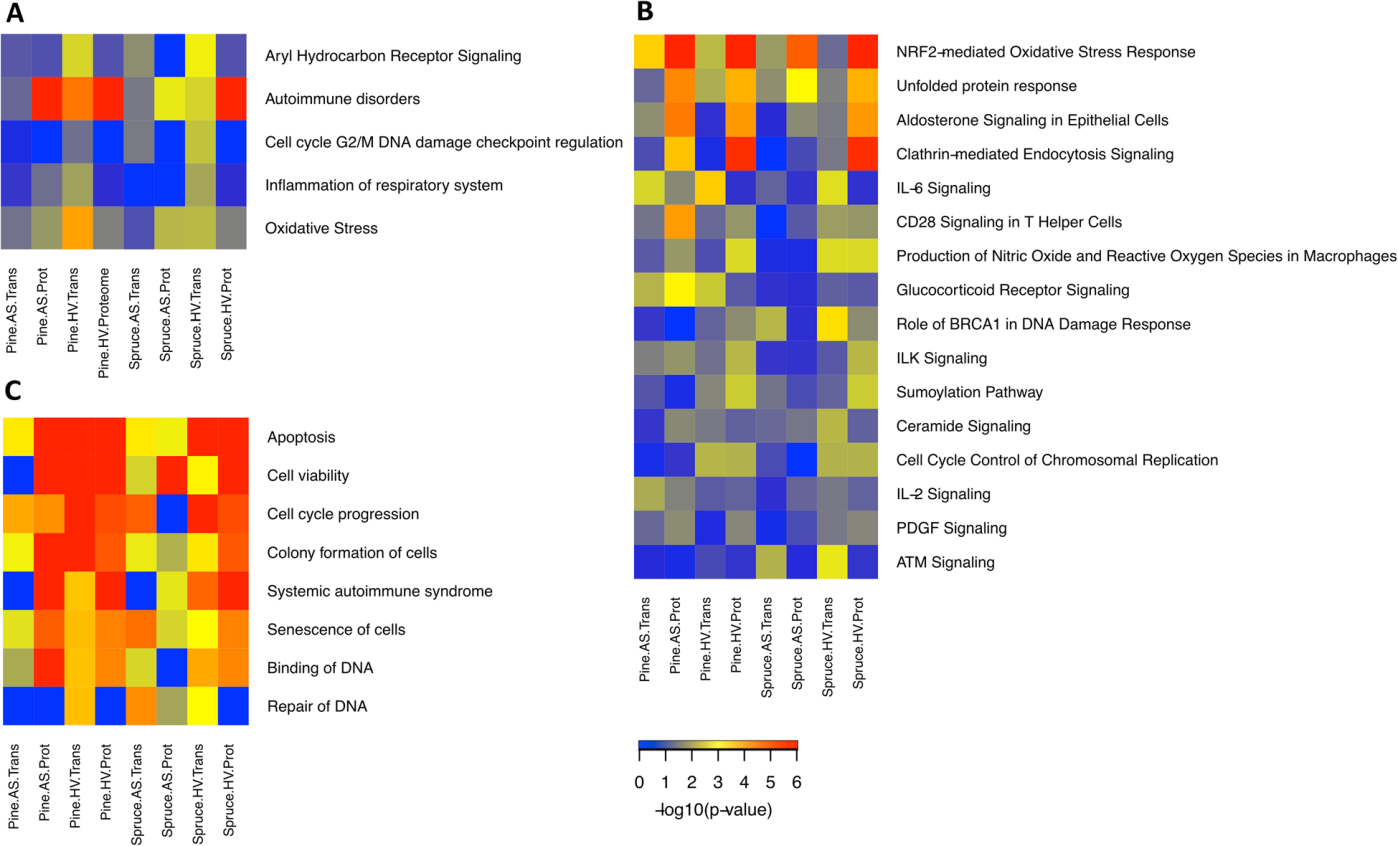
RAW264.7 cell IPA comparison analysis of biological processes **a**, canonical pathways **b** and diseases and biofunctions **c** of transcriptome and proteome data from both aerosol (AS) and aerosol enhanced deposition (HV) following pine and spruce exposure. Red indicates high, yellow intermediate and blue slight regulation

IPA of biological processes revealed several pathways to be significantly regulated at the transcriptome and proteome levels (Fig. 8). Aryl hydrocarbon receptor (AhR) signalling and cell cycle G2/M DNA damage checkpoint regulation were mainly regulated at the transcriptome level, while oxidative stress, autoimmune disorders and inflammation of the respiratory system were significantly regulated at both the transcriptome and proteome levels (Fig. 8a). The canonical pathway comparison showed common regulation at both the transcriptome and proteome levels for several pathways (Fig. 8b) involved in oxidative and cellular stress responses as well as immunomodulation. Additionally, pine combustion aerosol exposures were more effective in inducing glucocorticoid receptor signalling at the transcript and protein levels, while following spruce exposure, DNA damage signalling and repair were more significantly regulated. Consistently, differences between the wood CCA exposures were also highlighted when comparing IPA disease and biofunctions. This comparison shows that DNA damage and repair pathways were mostly induced by spruce combustion aerosols and not by pine exposure, at both the transcriptome and proteome level (Fig. 8c).

## Discussion

The aim of the study was to assess in vivo and in vitro toxicological effects, using three different ALI systems and mouse whole-body inhalation, after exposure to spruce and pine combustion emissions. In addition, we investigated the difference in the composition of the spruce and pine combustion emissions with concurrent online and offline characterization of the aerosols. Finally, we compared the differences in the effects of complete combustion aerosols (CCAs) and HEPA-filtered aerosols (HFAs) on human A549 cells. Due to the short period in which this study was performed, we focused not on a detailed explanation of the biological effects observed but rather on showing the utility of multiple model systems and exposure methods combined with a comprehensive endpoint analysis to highlight critical physicochemical characteristics of combustion aerosols and toxicity pathways for further studies.

### Viability and cytotoxicity

All combustion phases during each 4 h combustion experiment were included in the OMICS-ALI exposures and mouse inhalation exposures (Fig. 1). The Tox-ALI exposures were conducted in a 1 h period for both CCA exposure and HFA exposure. Different combustion phases affect emission characteristics and exhibit different toxicological outcomes [17, 24, 55]. In this study, the CCA exposures showed differences in the emissions from the combustion of different wood fuels. Notable differences emerged both between batches and between exposure days, supporting the notion that every combustion event is unique and that multiple factors affect combustion emissions, even in highly controlled experiments. In addition, combustion parameters in exposure aerosol are shown at the Table 1. They can be converted to emission factors using approximate emission conversion factor (ECF) given in the same table, allowing comparisons of the emissions with other combustion studies. Overall, this variation was in good agreement with previous studies [7, 9, 12, 56, 57].

PM mass, as well as PAH and o-PAH concentrations (Table 2 and Supplementary Table 2), differed considerably. Batches of both wood types weighed the same, and combustion experiments had very similar conditions. However, pine combustion produced a higher PM mass concentration in the emissions than spruce combustion. Spruce combustion produced, smaller particles on average, leading to overall similar PNCs in both wood fuels. These differences in emission characteristics are most likely responsible for the detected differences highlighted in the toxicological endpoints.

It has been previously shown that PM from biomass combustion damages cells [24, 32, 34]. In this study, the A549 and RAW264.7 cells from the OMICS-ALI exposure system exhibited cytotoxic responses following spruce and pine combustion emission exposure, especially with enhanced deposition (Fig. 4). After enhanced deposition of spruce CCA, A549 cells revealed higher cytotoxicity. Pine CCA exposure induced lower cytotoxicity, but the effect was still statistically significant. Batch differences most likely explain the clearly increased SD in spruce CCA-exposed samples, which showed higher toxicity than pine-exposed samples but not a statistically significant difference. The larger differences are probably due to the different burning characteristics of the two fuels. In addition, study from Mülhopt et al. [28] that used similar ALI system with enhanced deposition in attracting naturally charged PM onto the cells indicated that the electric field have no effect in viability and neither the usage of high voltage alters the exposure emission by producing by-products such as ozone. Ozone is known to be generated in some of the ALI systems that inhabits corona discharger in particle charging [58]. Particle charging allows PM to turn charged and to deposit PM into counter electrode collection plate [59]. Yet, using high voltage in one of our ALI system, there is no by-product formation as it was used only in attracting naturally charged particles and thus, there should not be effect on cytotoxicity. In Tox-ALI, the exposure was for 1 h, while in OMICS-ALI, it was 4 h. This resulted in similar deposited masses in both systems. Without electrostatic deposition enhancement, OMICS-ALI and Tox-ALI revealed a similar small-to-negligible effect on cell viability or cytotoxicity (Figs. 3a and 4a). Therefore, we suggest that the adverse effects on cells observed in the viability and cytotoxicity measurements were the result of higher amounts of deposited aerosols. However, the possibility that the electric field harmed the cells is not completely eliminated.

Mouse lung single-cell suspension results indicated that the pine combustion aerosol decreased cell viability more effectively than spruce combustion emissions (Fig. 5). Mice were exposed to CCA for 4 h on three consecutive days, and the measurements of cell viability in the BALF were high. Decreased viability was observed during these experiments only in single-cell suspensions from the lungs, but this decrease was also measured in untreated animals. It is possible that single-cell suspensions of lung homogenates showed lower viability in general due to the preparation process. Moreover, the deposited PM dose in the lungs was lower than in other studies [60, 61], indicating that further studies require more or longer exposures to achieve higher responses from mouse experiments (Table 3). To summarize, our aim was not to cause cell death per se but to investigate health-related effects at an exposure level high enough to trigger other cellular responses during similar short acute exposures in vitro and in vivo.

### Oxidative stress

Reactive oxygen species (ROS) formation is a cellular response seen in various combustion emission exposure studies [25, 29, 62, 63]. In this study, intracellular ROS formation was not detected by the 2′,7′-dichlorodihydrofluorescein diacetate (H_2_DCFDA) assay in Tox-ALI-exposed cells (Supplementary Materials and Supplementary Fig. 5, additional files 2 and 11). Unfortunately, this measurement was only taken after a subsequent 24-h sample incubation and any early ROS signal may have dissipated. This is supported by a previous mouse study with diesel exhaust particle exposure showing that the ROS level peaked at the 6 h timepoint but was below the control value at the 24 h timepoint [64].

Despite no measurement of direct ROS activity, transcriptome and proteome analysis revealed that exposure to emission aerosols induced the regulation of oxidative stress-related genes. Indeed, most of the upregulated pathways were related to enhanced oxidative stress following both spruce and pine CCA exposure. These results are in line with in vivo studies on wood combustion exposure in mice showing that oxidative stress is induced by exposure [64–66]. For instance, the NRF-2-mediated oxidative stress pathway was significantly regulated in both the transcript and proteome analyses after in vitro and in vivo combustion emission aerosol exposure. This activation could be caused by several compounds, including PAHs, transition metals or elemental carbon [33, 67]. In our experiment, all of the above mentioned factors were detected in the aerosols, indicating the possibility of ROS induction. Activation of the NRF-2 pathway could indicate a role of transitional metals since there is a known connection of transitional metals with haem-oxygenase-1 (HO-1), which is one of the targets of the NRF-2 pathway [68]. Li et al. [67] argued that transitional metals and elemental carbon together cause wood combustion PM to produce a stronger HO-1 response than coal combustion-derived PM. Moreover, the oxidative stress, production of nitric oxide and the ROS pathways were significantly upregulated at both the transcript and proteome levels in RAW264.7 cells (Table 5 and Figs. 7 and 8). In response to pine CCA, the cells showed induction of ATP biosynthetic processes and mechanisms regulating cell redox homeostasis.

Analysis of changes in the transcriptome, proteome, viability and intracellular ROS shows that the cells survived acute exposure to the aerosols without a notable decrease in viability. This survival response may have been achieved due to AhR signalling, which was more significantly activated following pine CCA exposure than spruce exposure. AhR is a ligand-activated transcription factor regulating cytochrome P450 (CYP) 1A1, 1A2 and 1B1, which metabolizes harmful substances to less toxic forms. Most importantly, AhR signalling is triggered by exogenous ligands, such as PAH compounds (O’Driscoll et al. [98]), which were more abundant in pine than in spruce combustion emissions. AhR has been previously shown to have some protective function against oxidative stress and apoptosis and is able to suppress inflammation caused by cigarette smoke [69–71]. The only exception with increased cytotoxicity was after enhanced deposition experiments, indicating a possible adverse effect of the electric field on the cells, which must be considered when comparing the enhanced deposition experiments to other experiments. Altogether, both transcriptomic and proteomic results indicate that notable levels of oxidative stress occurred in both exposed cells and animals. In addition, it is highly likely that oxidative stress has a role in the genotoxic and inflammatory responses that are discussed in the following sections.

### Genotoxicity

The clearest difference between the two wood combustion emissions was in the concentrations of PAH compounds, that were much more abundant in the pine combustion samples than in spruce (Table 2 and Supplementary Table 2). PAH compounds are well known to cause genotoxicity, oxidative stress and downregulation of neutrophils in the lungs [31, 72]. It has been reported that lighter PAH compounds can be in either the gaseous or particulate phase, whereas ≥4 ring PAH compounds are predominantly in the particulate phase [72]. To highlight a few individual PAH compounds from the high number of those measured in this study, phenanthrene, anthracene and cyclopenta [cd] pyrene had the highest difference between wood types, showing 5-fold higher concentrations in pine combustion emissions. However, when inspecting the toxic equivalence (TEQ), the highest difference between wood types was seen for dibenzo [al] pyrene (27-fold), benzo [a] pyrene (6.6-fold) and dibenzo [ai] pyrene (3.6-fold), which was expected due to their higher TEF value. For o-PAHs, 9H-fluoren-9-one (3.2-fold) and 1H-phenalen-1-one (2.1-fold) had large differences between wood types, with two- to three-fold higher concentrations in pine combustion emission than in spruce. Although 1H-phenalen-1-one (perinaphthenone) seems to be less mutagenic than benzo [a] pyrene [73], it was more than 40 times higher in concentration.

A549 cells revealed DNA damage following CCA and HFA exposure, but spruce CCA caused the highest but not statistically significant amount of DNA damage in cells. This was contradictory to the mouse measurements where pine combustion aerosol exposure was more potent in causing DNA damage such as double strand breaks (Fig. 3b and Fig. 5). Moreover, both CCA and HFA caused equal genotoxicity in A549 cells after pine combustion aerosol exposure. Similar results were also observed after spruce HFA exposure, with CCA causing a smaller response. Altogether, this could indicate that the induced DNA damage originates mostly from gaseous compounds and not predominantly from PM. This was supported by HFA exposure, where the PM was removed by HEPA filtration, and the same outcome was observed as for pine CCA exposure. Pine CCA emissions had larger particle sizes and higher mass concentrations than the corresponding spruce combustion aerosols, which may have somewhat affected the genotoxicity.

It is known that gaseous PAHs, carbonyls, and other gaseous compounds such as NO_2_ can generate DNA damage in cells either directly or indirectly, for example, by oxidative stress [74–76]. Based on our results, we suggest that gaseous molecules should be given more attention in future studies. Many studies (e.g., [9, 77, 78]) have focused only on PM and therefore indicated that the causative agents for genotoxic responses originate from PM and compounds bound to it. Moreover, Zou et al. [79] suggest that pine 95% of genotoxic PAHs are bound to PM. Interestingly, spruce CCA exposure showed the largest DNA damage potential despite the emissions having three-fold lower amounts of PAH compounds and both lower PM_1_ mass and number concentrations compared to the corresponding pine emissions. If the PAH compounds alone caused the observed DNA damage, pine CCA exposure should have caused higher responses than spruce exposure, which was not the case here.

It remains unclear why pine emission did not show the stronger genotoxic effects suggested by the higher PAH concentrations and deposited PM mass. It could be that the DNA damaging components of spruce emission PM were more easily reactive with the cells than those in pine combustion PM. Smaller particle size could be one of the explanations behind this difference. To cause genotoxic effects, PM must cross the cell membrane and react inside the cell to create ROS, which in turn cause DNA damage after several steps [80]. A549 cells were left to react with deposited PM for 24 h after Tox-ALI exposure, and during this time, the active components of PM should have been able to react with the cells. PM size may have had a role in how the particle-bound components have been able to enter the cells in our study.

The effects of PAH and PM concentration differences were perhaps more logical in mouse exposures, where the pine combustion aerosols showed both the highest PAH concentrations and the largest level of DNA damage in both BALF and single-cell suspensions of the lungs (Fig. 5b and c). In the BALF, pine CCA-exposed samples showed two-fold higher amounts of DNA damage than the corresponding spruce combustion aerosol samples. However, less of a difference was observed in single-cell suspensions of lungs. It could be that the cells collected in BALF are the first cells to be in contact with aerosols, hence reacting with the aerosols for more time and with higher concentrations. This would allow a higher proportion of BALF cells to be affected by aerosols in comparison to lung tissue. In our previous study in which we compared different stoves and pellet boilers, a slight increase in DNA damage in BALF was observed after the intratracheal instillation of wood smoke particles [13]. The increase in DNA damage was hypothesized to be caused by the PM-induced generation of intracellular ROS. However, another study with higher PM amounts than this study showed no genotoxic effect and a low intracellular ROS signal [61].

The genotoxic results from cells and mice are supported by the transcript and proteome measurements showing that pine combustion emissions were more effective in vitro, but not in vivo, in activating the Nrf2 pathway. Thus, cells in vitro controlled redox homeostasis and related DNA repair processes better (Table 5 and Fig. 8). This activation could help the cells overcome some of the DNA damage, as evidenced by the lower level of damaged DNA in cells exposed to pine combustion emission compared to spruce. In addition, the mouse exposure lasting three consecutive days could be enough to overcome the repair system capability and cause DNA damage. One other possibility could be that pine combustion PM contains more protective agents against oxidative reactions than spruce combustion PM. Kjällstrand and Petersson [81] suggested that antioxidants such as phenolic compounds in wood smoke could decrease the carcinogenic effects of PAH compounds. Unfortunately, our study did not measure these compounds, and the antioxidant capabilities of the PM and the gases need to be assessed in future studies.

### Inflammatory response

In our study, inorganic compounds were found at notable levels at the emissions of both wood types (Supplementary Table 3). Among the chemical components of wood combustion emission, inorganic metals such as Cd, Cu, Mg, Mn and Zn are known to induce inflammatory responses [35, 82–85]. The highest difference between the two combustion emissions was observed in Cu and Zn concentrations that were two- to three-fold higher in spruce than in the respective pine emissions. In a study by Rice et al. [83], doses of 0.1 and 1 μmol kg^− 1^ Cu were seen to cause large effects by inducing inflammatory response and cytotoxicity, whereas similar amounts of Zn resulted in a much lower level of association between composition and inflammatory response. Zn has been shown in other studies [84, 85] to have a larger role in inducing cytotoxicity and proinflammatory responses than was shown in Rice et al. [83]. In vitro studies by Riley et al. [84] and Uski et al. [85] concluded that Zn must be in a soluble form to cause the observed harmful effects. Based on the levels of inorganic elements measured from combustion emissions of both wood types, the inflammatory responses could have been expected to be higher than those observed in this study. However, in the complex mixture, the result is always the sum of different compounds that may have opposite effects. During our exposure and post-exposure incubation, the PM-bound Zn should be able to become soluble under the conditions used in the in vitro and in vivo experiments. The inflammatory responses measured in our study could have been suppressed by high PAH concentrations since PAH components have been shown in various studies to be immunosuppressive [72, 86, 87]. The immunosuppressive effects of PAHs could possibly increase up to a certain PAH threshold concentration beyond which no further effects are observed. However, it could also be that the cumulative concentrations in the lungs were not high enough to cause an inflammatory response.

A549 cells exposed to combustion aerosols in Tox-ALI showed mildly and nonsignificantly elevated cytokine levels (Fig. 3c); however, HFA-exposed cells showed a slightly higher nonsignificant inflammatory response than CCA-exposed cells suggesting that the gaseous fraction of the emissions had larger effects than expected. The somewhat different combustion emissions during CCA and HFA measurement may have translated into different inflammatory responses, but the role of gaseous vs. PM components cannot be eliminated. IL-8 (an analogue of mouse KC) secretion showed no notable differences between the two wood fuel types in the A549 cells. However, cytokine measurement did show a slight difference between CCA- and HFA-exposed cells, indicating that components other than PM-bound components induced the majority of inflammatory responses. The likely candidates are gaseous components, including volatile organic compounds as well as NO_x_ and SO_x_ [88–90]. It is possible that PAH compounds in the particulate and gaseous phases could harm the normal metabolism of the cells and consequently suppress IL-8 secretion. This effect might be greater in CCA-exposed cells than in HFA-exposed cells. Interestingly, pine combustion emission contained more PAH compounds than spruce combustion emission, yet the secreted IL-8 concentrations were at the same level in both conditions. During CCA exposures, much higher PM peaks were detected in the emission aerosol than during HFA exposures before filtering, which may have increased the proportion of PAHs bound to PM during those experiments, causing the PAHs to be filtered from the emission with the particles. It is also possible that during HFA exposure, there may have been more gaseous PAHs in the emission aerosols.

In our experiments, we detected overall low cytokine levels in mice. However, significant changes in these low levels were seen in both BALF and serum as a result of exposure (Table 4). Previous studies with mice, rats and humans are in line with our in vivo findings of low inflammatory responses to wood combustion emission [34, 35, 91–93]. In the spruce CCA-exposed mice, the concentrations of IL-6, TNFα and KC in the BALF were two- to three-fold higher than those in untreated mice. In contrast, in the BALF of pine CCA-exposed mice, these cytokines were either at the same level or only slightly increased compared to untreated controls. Among these cytokines, IL-6 and KC also showed a two-fold difference between emission aerosols from the combustion of two wood types, and the responses to spruce emission were higher. This could be explained by the different ratios of organic (e.g., PAHs) and inorganic (e.g., metals) concentrations in these two emission aerosols. The levels of these two cytokines in the serum samples were at the same level as in untreated mice. The concentration of IL-6 measured from BALF of the spruce CCA-exposed mice was two-fold higher than in the respective pine CCA-exposed mice and unexposed controls. IL-6 is known to induce acute phase protein synthesis under the regulation of TNFα [24]. Therefore, TNFα was expected to reflect the increase in IL-6 level, which could indicate active early inflammatory responses. In contrast, after exposure to pine CCA, the early inflammatory response accompanied by an increase in TNFα release could have peaked earlier and possibly overcompensated by feedback mechanisms during the time of measurement, as indicated by the significantly lower serum level of TNFα compared to that in untreated mice.

Transcriptome and proteome data showed that the acute inflammatory response controlled by TREM1 signalling was more significantly induced in vivo by spruce CCA exposure than by pine exposure. Induced immune responses in terms of agranulocyte infiltration and crosstalk between innate and adaptive immune systems highlighted different immunological responses between the spruce and pine exposures in the omics analyses. Although the inflammatory response was mildly induced overall by pine CCA, peroxisome proliferator-activated receptor (PPAR) signalling was significantly upregulated following all in vitro pine combustion aerosol exposures as well as after enhanced deposition in spruce combustion aerosol exposures of cells. The PPAR signalling pathway plays an important role in several cellular processes, including inflammation and immune responses, by reducing the influx of inflammatory cells and cytokine production [94]. In addition, asthma, a known inflammatory disease, is known to worsen upon exposure to PM, and one gene, *SYNE1*, has been suggested to have a role in asthma attacks [54, 95]. In this study, the *SYNE1* gene was similarly regulated between wood types but oppositely regulated in human cells and mouse cells. This gene was upregulated in A549 cells but downregulated in both RAW267.4 cells and mouse BALF cells. The *SYNE1* gene provides instructions for a linking network between organelles and the actin cytoskeleton to establish and maintain the subcellular spatial organization as well as the subcellular nuclei positions in structured tissues [96].

## Conclusions

Our findings showed that concurrent in vitro and in vivo exposures present different responses and indicate new aspects of the interaction of combustion aerosols in different exposure systems. Systems biology approaches revealed that the Nrf2 pathway was much more strongly activated in vitro than in vivo at both the transcriptome and proteome levels, showing differences in the oxidative stress pathways between the used models. Thus, our study supports parallel experiments performed in vitro and in vivo.

This study was done to shape future aerosol studies since the comparability of in vivo and in vitro results is not yet addressed as extensively as it should be. At the same time, cell-based methods have become preferred as alternatives to animal experiments. Establishing the relevance of in vitro findings requires a better understanding of their equivalence to in vivo results. We have proven here that there are major differences in responses to the exposed aerosols as examined by in vivo and in vitro methods. There is more to unravel regarding the cellular interactions to enable analysis of the outcome of aerosol exposure based on in vitro and in vivo studies alone.

## Supplementary information

**Additional file 1: Table S1.** Calibration and quantification mixtures of isotope labelled internal standards and calibration standards.

**Additional file 2.** Supplementary materials and methods

**Additional file 3 Figure S1.** Physical properties in the diluted exposure exhaust per day to day, for spruce (A) and pine (B) experiments. Parts A and C show average of 3 experiment days total suspended particulate mass (TSP), lung deposited surface area (LDSA) and particle number concentration (PNC) during exposures.

**Additional file 4 Figure S2.** Size of the particles measured by SMPS per day to day. The geometric mean diameters are shown with the solid lines, while GSDs are represented with dashed lines. (average of 3 experiments)

**Additional file 5 Figure S3.** Size of the particles measured by SMPS. The geometric mean diameters (a) and mass median diameters (b) are shown with the solid lines, while GSDs are represented with dashed lines. Average of 3 experiments.

**Additional file 6 Table S2.** All measured organic compounds from wood combustion emissions. Organic compounds were sampled over 4-h exposure. Concentrations are calculated as averages from three test cycles ± standard deviation.

**Additional file 7 Table S3.** Inorganic elements from spruce and pine combustion emissions. Organic compounds were sampled over 4-h exposure. Concentrations are calculated as averages from three test cycles ± standard deviation.

**Additional file 8 Figure S4.** Viability (A), Comet assay (B) and IL-8 secretion (C) of A549 control cells after exposed one hour at incubator with CO2, incubator without CO2 or Tox-ALI to clean air. Each bar shows mean ± SEM, *n* = 3.

**Additional file 9 Table S4.** Top 5 significant ingenuity canonical pathways of spruce aerosol exposure.

**Additional file 10 Table S5.** Top 5 significant ingenuity canonical pathways of pine aerosol exposed cells.

**Additional file 11 Figure S5.** Area under curve (AUC) calculated from exposed A549 cells from three different timepoints of H2DCFDA measurements. Each bar shows mean ± SEM, *n* = 3.

## Data Availability

The datasets supporting the conclusions of this article are included within the article (and its additional files).

## Abbreviations

H_2_DCFDA: 2′,7′-dichlorodihydrofluorescein diacetate
DAPI: 4′,6-diamidino-2-phenylindole
FTIR: A Fourier Transform Infrared Analyzer
AhR: Aryl Hydrocarbon Receptor
BALF: Bronchoalveolar lavage fluid
CCA: Complete combustion aerosol
CYP: Cytochrome P450
DMEM: Dulbecco’s Modified Eagle Medium
ELPI: Electrical Low-Pressure Impactor
HV: High voltage
FBS: Fetal bovine serum
GO: Gene ontology
GMD: Geometric mean diameter
HFA: HEPA filtered aerosol
HEPA: High efficient particulate air
A549: Human alveolar epithelial cell line
IPA: Ingenuity Pathway Analysis
IDTD-GC-ToFMS: In-situ derivatization and thermal desorption - gas-chromatography - time-of-flight mass spectrometry
LDSA: Lung deposited surface area
MMD: Mass median diameter
RAW264.7: Murine macrophages cell lines
NSAM: Nanoparticle Surface Area Monitor
o-PAH: Oxygenated derivatives of PAH
PNC: Particle number concentrations
PM_1_: Particulate matter < 1 μm
PPAR: Peroxisome proliferator-activated receptors
PBS: Phosphate-buffered saline
QFF: Quartz fiber filter
RPMI: Roswell Park Memorial Institute
TSP: Total suspended particulate mass
TEF: Toxic equivalency factor
TEQ: Toxic equivalent
TEAB: Tri-ethyl ammonium bicarbonate
EDTA: Trypsin-ethylenediaminetetraacetic acid

## References

[1] Cohen AJ, Brauer M, Burnett R, Anderson HR, Frostad J, Estep K, Balakrishnan K, Brunekreef B, Dandona L, Dandona R (2017). Estimates and 25-year trends of the global burden of disease attributable to ambient air pollution: an analysis of data from the global burden of diseases study 2015. Lancet.

[2] Silva RA, Adelman Z, Fry MM, West JJ (2016). The impact of individual anthropogenic emissions sectors on the global burden of human mortality due to ambient air pollution. Environ Health Perspect.

[3] Knorr W, Dentener F, Lamarque JF, Jiang L, Arneth A (2017). Wildfire air pollution hazard during the 21st century. Atmos Chem Phys.

[4] Vicente ED, Alves CA (2018). An overview of particulate emissions from residential biomass combustion. Atmos Res.

[5] Sigsgaard T, Forsberg B, Annesi-Maesano I, Blomberg A, Bølling A, Boman C, Bønløkke J, Brauer M, Bruce N, Héroux M, Hirvonen M, Kelly F, Künzli N, Lundbäck B, Moshammer H, Noonan C, Pagels J, Sallsten G, Sculier J, Brunekreef B (2015). Health impacts of anthropogenic biomass burning in the developed world. Eur Respir J.

[6] Torvelainen, J. Pientalojen polttopuun käyttö 2016/2017. https://stat.luke.fi/pientalojen-polttopuun-k%C3%A4ytt%C3%B6-20162017_fi. Accessed 28 Aug 2019.

[7] Czech H, Miersch T, Orasche J, Abbaszade G, Sippula O, Tissari J, Michalke B, Schnelle-Kreis J, Streibel T, Jokiniemi J, Zimmermann R (2018). Chemical composition and speciation of particulate organic matter from modern residential small-scale wood combustion appliances. Sci Total Environ.

[8] Gminski R, Tang T, Mersch-Sundermann V (2010). Cytotoxicity and genotoxicity in human lung epithelial A549 cells caused by airborne volatile organic compounds emitted from pine wood and oriented strand boards. Toxicol Lett.

[9] Kasurinen S, Jalava PI, Happo MS, Sippula O, Uski O, Koponen H, Orasche J, Zimmermann R, Jokiniemi J, Hirvonen M (2017). Particulate emissions from the combustion of birch, beech, and spruce logs cause different cytotoxic responses in A549 cells. Environ Toxicol.

[10] Kistler M, Schmidl C, Padouvas E, Giebl H, Lohninger J, Ellinger R, Bauer H, Puxbaum H (2012). Odor, gaseous and PM 10 emissions from small scale combustion of wood types indigenous to Central Europe. Atmos Environ.

[11] Orasche J, Seidel T, Hartmann H, Schnelle-Kreis J, Chow JC, Ruppert H, Zimmermann R (2012). Comparison of emissions from wood combustion. Part 1: emission factors and characteristics from different small-scale residential heating appliances considering particulate matter and polycyclic aromatic hydrocarbon (PAH)-related toxicological potential of particle-bound organic species. Energ Fuel.

[12] Orasche J, Schnelle-Kreis J, Schön C, Hartmann H, Ruppert H, Arteaga-Salas JM, Zimmermann R (2013). Comparison of emissions from wood combustion. Part 2: impact of combustion conditions on emission factors and characteristics of particle-bound organic species and polycyclic aromatic hydrocarbon (PAH)-related toxicological potential. Energ Fuel..

[13] Happo MS, Uski O, Jalava PI, Kelz J, Brunner T, Hakulinen P, Mäki-Paakkanen J, Kosma V, Jokiniemi J, Obernberger I, Hirvonen M (2013). Pulmonary inflammation and tissue damage in the mouse lung after exposure to PM samples from biomass heating appliances of old and modern technologies. Sci Total Environ.

[14] Jalava PI, Happo MS, Kelz J, Brunner T, Hakulinen P, Mäki-Paakkanen J, Hukkanen A, Jokiniemi J, Obernberger I, Hirvonen M.-. (2012). Invitro toxicological characterization of particulate emissions from residential biomass heating systems based on old and new technologies. Atmos Environ.

[15] McDonald JD, Zielinska B, Fujita EM, Sagebiel JC, Chow JC, Watson JG (2000). Fine particle and gaseous emission rates from residential wood combustion. Environ Sci Technol.

[16] Schmidl C, Luisser M, Padouvas E, Lasselsberger L, Rzaca M, Ramirez-Santa Cruz C, Handler M, Peng G, Bauer H, Puxbaum H (2011). Particulate and gaseous emissions from manually and automatically fired small scale combustion systems. Atmos Environ.

[17] Tissari J, Lyyränen J, Hytönen K, Sippula O, Tapper U, Frey A, Saarnio K, Pennanen AS, Hillamo R, Salonen RO, Hirvonen M.-. & Jokiniemi J. (2008). Fine particle and gaseous emissions from normal and smouldering wood combustion in a conventional masonry heater. Atmos Environ.

[18] Weggler BA, Ly-Verdu S, Jennerwein M, Sippula O, Reda AA, Orasche J, Gröger T, Jokiniemi J, Zimmermann R (2016). Untargeted identification of wood type-specific markers in particulate matter from wood combustion. Environ Technol.

[19] Barregard L, Sällsten LG, Andersson L, Almstrand A-C, Gustafson P, Andersson M.& Olin A.-C. (2008). Experimental exposure to wood smoke: effects on airway inflammation and oxidative stress. Occup Environ Med.

[20] Dogan OT, Elagoz S, Ozsahin SL, Epozturk K, Tuncer E, Akkurt I (2011). Pulmonary toxicity of chronic exposure to tobacco and biomass smoke in rats. Clinics..

[21] Ocakli B, Acarturk E, Aksoy E, Gungor S, Ciyiltepe F, Oztas S, Ozmen I, Agca MC, Salturk C, Adiguzel N, Karakurt Z (2018). The impact of exposure to biomass smoke versus cigarette smoke on inflammatory markers and pulmonary function parameters in patients with chronic respiratory failure. Int J Chron Obstruct Pulmon Dis.

[22] Thompson J (2018). Airborne particulate matter: human exposure and health effects. J Occup Environ Med.

[23] Ramos C, Pedraza-Chaverri J, Becerril C, Cisneros J, González-Ávila G, Rivera-Rosales R, Sommer B, Medina-Campos ON, Montaño M (2013). Oxidative stress and lung injury induced by short-term exposure to wood smoke in Guinea pigs. Toxicol Mech Methods.

[24] Bølling AK, Totlandsdal AI, Sällsten G, Braun A, Westerholm R, Bergvall C, Boman J, Dahlman HJ, Sehlstedt M, Cassee F, Sandstrom T, Schwarze PE, Herseth JI (2012). Wood smoke particles from different combustion phases induce similar pro-inflammatory effects in a co-culture of monocyte and pneumocyte cell lines. Part Fibre Toxicol..

[25] Dilger M, Orasche J, Zimmermann R, Paur H, Diabaté S, Weiss C (2016). Toxicity of wood smoke particles in human A549 lung epithelial cells: the role of PAHs, soot and zinc. Arch Toxicol.

[26] Aufderheide M, Mohr U (1999). CULTEX — a new system and technique for the cultivation and exposure of cells at the air/liquid interface. Exp Toxicol Pathol.

[27] Ihalainen M, Jalava P, Ihantola T, Kasurinen S, Uski O, Sippula O, Hartikainen A, Tissari J, Kuuspalo K, Lähde A, Hirvonen M, Jokiniemi J (2019). Design and validation of an air-liquid interface (ALI) exposure device based on thermophoresis. Aerosol Sci Technol.

[28] Mülhopt S, Dilger M, Diabaté S, Schlager C, Krebs T, Zimmermann R, Buters J, Oeder S, Wäscher T, Weiss C, Paur H (2016). Toxicity testing of combustion aerosols at the air–liquid interface with a self-contained and easy-to-use exposure system. J Aerosol Sci.

[29] Danielsen PH, Moller P, Jensen KA, Sharma AK, Wallin H, Bossi R, Autrup H, Molhave L, Ravanat J, Briede JJ, de Kok TM, Loft S (2011). Oxidative stress, DNA damage, and inflammation induced by ambient air and wood smoke particulate matter in human A549 and THP-1 cell lines. Chem Res Toxicol.

[30] Kasurinen S, Happo MS, Rönkkö TJ, Orasche J, Jokiniemi J, Kortelainen M, Tissari J, Zimmermann R, Hirvonen M, Jalava PI (2018). Differences between co-cultures and monocultures in testing the toxicity of particulate matter derived from log wood and pellet combustion. PLoS One.

[31] Kocbach A, Namork E, Schwarze PE (2008). Pro-inflammatory potential of wood smoke and traffic-derived particles in a monocytic cell line. Toxicology..

[32] Landkocz Y, Ledoux F, André V, Cazier F, Genevray P, Dewaele D, Martin PJ, Lepers C, Verdin A, Courcot D, Courcot L, Boushina S, Sichel F, Gualtieri M, Shirali P, Billet S (2017). Fine and ultrafine atmospheric particulate matter at a multi-influenced urban site: physicochemical characterization, mutagenicity and cytotoxicity. Environ Pollut.

[33] Uski O, Jalava PI, Happo MS, Leskinen J, Sippula O, Tissari J, Mäki-Paakkanen J, Jokiniemi J, Hirvonen M-R (2014). Different toxic mechanisms are activated by emission PM depending on combustion efficiency. Atmos Environ.

[34] Muala A, Rankin G, Sehlstedt M, Unosson J, Bosson JA, Behndig A, Pourazar J, Nyström R, Pettersson E, Bergvall C, Westerholm R, Jalava PI, Happo MS, Uski O, Hirvonen M, Kelly FJ, Mudway IS, Blomberg A, Boman C, Sandström T (2015). Acute exposure to wood smoke from incomplete combustion - indications of cytotoxicity. Part. Fibre. Toxicol..

[35] Uski OJ, Happo MS, Jalava PI, Brunner T, Kelz J, Obernberger I, Jokiniemi J, Hirvonen M (2012). Acute systemic and lung inflammation in C57Bl/6J mice after intratracheal aspiration of particulate matter from small-scale biomass combustion appliances based on old and modern technologies. Inhal Toxicol.

[36] Chow JC, Watson JG, Chen L-A, Chang MCO, Robinson NF, Trimble D, Kohl S (2007). The IMPROVE_A temperature protocol for thermal/optical carbon analysis: maintaining consistency with a long-term database. J Air Waste Manage Assoc.

[37] Orasche J, Schnelle-Kreis J, Abbaszade G, Zimmermann R (2011). Technical note: in-situ derivatization thermal desorption GC-TOFMS for direct analysis of particle-bound non-polar and polar organic species. Atmos Chem and Phys.

[38] Oeder S, Kanashova T, Sippula O, Sapcariu SC, Streibel T, Arteaga-Salas JM, Passig J, Dilger M, Paur H, Schlager C, Mulhopt S, Diabate S, Weiss C, Stengel B, Rabe R, Harndorf H, Torvela T, Jokiniemi JK, Hirvonen M, Schmidt-Weber C, Traidl-Hoffmann C, BéruBé KA, Wlodarczyk AJ, Prytherch Z, Michalke B, Krebs T, Prévôt ASH, Kelbg M, Tiggesbäumker J, Karg E, Jakobi G, Scholtes S, Schnelle-Kreis J, Lintelmann J, Matuschek G, Sklorz M, Klingbeil S, Orasche J, Richthammer P, Müller L, Elsasser M, Reda A, Gröger T, Weggler B, Schwemer T, Czech H, Rüger CP, Abbaszade G, Radischat C, Hiller K, Buters JTM, Dittmar G, Zimmermann R (2015). Particulate matter from both heavy fuel oil and diesel fuel shipping emissions show strong biological effects on human lung cells at realistic and comparable in vitro exposure conditions. PLoS One.

[39] Sapcariu SC, Kanashova T, Dilger M, Diabaté S, Oeder S, Passig J, Radischat C, Buters J, Sippula O, Streibel T, Paur H, Schlager C, Mülhopt S, Stengel B, Rabe R, Harndorf H, Krebs T, Karg E, Gröger T, Weiss C, Dittmar G, Hiller K, Zimmermann R (2016). Metabolic Profiling as Well as Stable Isotope Assisted Metabolic and Proteomic Analysis of RAW 264.7 Macrophages Exposed to Ship Engine Aerosol Emissions: Different Effects of Heavy Fuel Oil and Refined Diesel Fuel. PLoS One.

[40] John-Schuster G, Hager K, Conlon TM, Irmler M, Beckers J, Eickelberg O, Yildirim AÖ (2014). Cigarette smoke-induced iBALT mediates macrophage activation in a B cell-dependent manner in COPD. Am J Physiol Lung Cell Mol Physiol.

[41] Paur H, Mülhopt S, Weiss C, Diabaté S (2008). In vitro exposure systems and bioassays for the assessment of toxicity of nanoparticles to the human lung. J Verbrauch Lebensm.

[42] Tippe A, Heinzmann U, Roth C (2002). Deposition of fine and ultrafine aerosol particles during exposure at the air/cell interface. J Aerosol Sci.

[43] Krebs, T. 2012. Automated exposure systems. Accessed march 23rd, 2015. http://www.vitrocell.com/inhalation-toxicology/exposure-systems/automated-exposure-station.

[44] Paur H, Cassee FR, Teeguarden J, Fissan H, Diabate S, Aufderheide M, Kreyling WG, Hänninen O, Kasper G, Riediker M, Rothen-Rutishauser B, Schmid O (2011). In-vitro cell exposure studies for the assessment of nanoparticle toxicity in the lung—A dialog between aerosol science and biology. J Aerosol Sci.

[45] Stone KC, Mercer RR, Gehr P, Stockstill B, Crapo JD (1992). Allometric relationships of cell numbers and size in the mammalian lung. Am J Respir Cell Mol Biol.

[46] Winkler-Heil R, Hofmann W (2016). Modeling particle deposition in the Balb/c mouse respiratory tract. Inhal Toxicol.

[47] Leskinen J, Ihalainen M, Torvela T, Kortelainen M, Lamberg H, Tiitta P, Jakobi G, Grigonyte J, Joutsensaari J, Sippula O, Tissari J, Virtanen A, Zimmermann R, Jokiniemi J (2014). Effective density and morphology of particles emitted from small-scale combustion of various wood fuels. Environ Technol.

[48] Hinds, W.C. 2012, Aerosol technology: properties, behavior, and measurement of airborne particles, John Wiley & Sons.

[49] Lenz AG, Karg E, Lentner B, Dittrich V, Brandenberger C, Rothen-Rutishauser B, Schulz H, Ferron GA, Schmid O (2009). A dose-controlled system for air-liquid interface cell exposure and application to zinc oxide nanoparticles. Part Fibre Toxicol..

[50] Jalava PI, Tapanainen M, Kuuspalo K, Markkanen A, Hakulinen P, Happo MS, Pennanen AS, Ihalainen M, Yli-Pirilä P, Makkonen U, Teinilä K, Mäki-Paakkanen J, Salonen RO, Jokiniemi J, Hirvonen M.-. (2010). Toxicological effects of emission particles from fossil- and biodiesel-fueled diesel engine with and without DOC/POC catalytic converter. Inhal Toxicol.

[51] Lam DCL, Chan SCH, Mak JCW, Freeman C, Ip MSM, Shum DKY (2015). S-maltoheptaose targets syndecan-bound effectors to reduce smoking-related neutrophilic inflammation. Sci Rep.

[52] Lee H, Jung K, Lee H, Park S, Choi W, Bae H (2001). International immunopharmacology. Int Immunopharmacol.

[53] Lee H, Jung K, Park S, Kil Y, Chung EY, Jang YP, Seo E, Bae H (2014). Inhibitory effects of Stemona tuberosa on lung inflammation in a subacute cigarette smoke-induced mouse model. BMC Complement Altern Med.

[54] Bérubé, J.C., Gaudreault, N., Lavoie-Charland, E., Sbarra, L., Henry, C., Madore, A.M., Paré, P.D., Van Den Berge, M., Nickle, D., Laviolette, M., Laprise, L., Bossé, Y,. 2016. Identification of susceptibility genes of adult asthma in French Canadian women. Can. Respir J. 10.1155/2016/3564341 PMC4904514 27445529

[55] Kim YH, Warren SH, Krantz QT, King C, Jaskot R, Preston WT, George BJ, Hays MD, Landis MS, Higuchi M, DeMarini DM, Gilmour MI (2018). Mutagenicity and Lung Toxicity of Smoldering vs Flaming Emissions from Various Biomass Fuels: Implications for Health Effects from Wildland Fires. Environ Health Perspect.

[56] Kortelainen M, Jokiniemi J, Tiitta P, Tissari J, Lamberg H, Leskinen J, Grigonyte-Lopez R, Koponen H, Antikainen S, Nuutinen I, Zimmermann R, Sippula O (2018). Time-resolved chemical composition of small-scale batch combustion emissions from various wood species. Fuel.

[57] Nuutinen K, Jokiniemi J, Sippula O, Lamberg H, Sutinen J, Horttanainen P, Tissari J (2014). Effect of air staging on fine particle, dust and gaseous emissions from masonry heaters. Biomass Bioenergy.

[58] De Bruijne K, Ebersviller S, Sexton KG, Lake S, Leith D, Goodman R, Jetters J, Walters GW, Doyle-Eisele M, Woodside R, Jefferies H, Jaspers I (2009). Design and testing of electrostatic aerosol in vitro exposure system (EAVES): an alternative exposure system for particles. Inhal Toxicol.

[59] Yu Z, Jang M, Sabo-Attwood T, Robinson SE, Jiang H (2017). Prediction of delivery of organic aerosols onto air-liquid Interface cells in vitro using an electrostatic precipitator. Toxicol in Vitro.

[60] Gerlofs-Nijland ME, Bokkers BGH, Sachse H, Reijnders JJE, Gustafsson M, Boere AJF, Fokkens PFH, Leseman DLAC, Augsburg K, Cassee FR. Inhalation toxicity profiles of particulate matter: a comparison between brake wear with other sources of emission. Inhal Toxicol. 2019;1-10. 10.1080/08958378.2019.1606365 31066325

[61] Skovmand A, Damiao Gouveia AC, Koponen IK, Møller P, Loft S, Roursgaard M (2017). Lung inflammation and genotoxicity in mice lungs after pulmonary exposure to candle light combustion particles. Toxicol Lett.

[62] Cho H, Park C, Shin H, Park K, Lim H (2018). Comparison of the in vitro toxicological activity of various particulate matter. Toxicol Ind Health.

[63] Torvela T, Uski O, Karhunen T, Lähde A, Jalava P, Sippula O, Tissari J, Hirvonen M, Jokiniemi J (2014). Reference particles for toxicological studies of wood combustion: formation, characteristics, and toxicity compared to those of real wood combustion particulate mass. Chem Res Toxicol.

[64] Zheng X, Wang G, Bin P, Meng T, Niu Y, Yang M, Zhang L, Duan H, Yu T, Dai Y, Zheng Y (2019). Time-course effects of antioxidants and phase II enzymes on diesel exhaust particles-induced oxidative damage in the mouse lung. Toxicol Appl Pharmacol.

[65] Loret T, Rogerieux F, Trouiller B, Braun A, Egles C, Lacroix G (2018). Predicting the in vivo pulmonary toxicity induced by acute exposure to poorly soluble nanomaterials by using advanced in vitro methods. Part Fibre Toxicol..

[66] Wegesser TC, Pinkerton KE, Last JA (2009). California wildfires of 2008: coarse and fine particulate matter toxicity. Environ Health Perspec.

[67] Li N, Champion WM, Imam J, Sidhu D, Salazar JR, Majestic BJ, Montoya LD (2018). Evaluation of cellular effects of fine particulate matter from combustion of solid fuels used for indoor heating on the Navajo nation using a stratified oxidative stress response model. Atmos Environ.

[68] Chang Y, Siddens LK, Heine LK, Sampson DA, Yu Z, Fischer KA, Löhr CV, Tilton SC. Comparative mechanisms of PAH toxicity by benzo [a] pyrene and dibenzo [def,p] chrysene in primary human bronchial epithelial cells cultured at air-liquid interface. Toxic Appl Pharm. 2019:379. 10.1016/j.taap.2019.114644 PMC6708476 31255691

[69] Baglole CJ, Maggirwar SB, Gasiewicz TA, Thatcher TH, Phipps RP, Sime PJ (2008). The aryl hydrocarbon receptor attenuates tobacco smoke-induced cyclooxygenase-2 and prostaglandin production in lung fibroblasts through regulation of the NF-kappaB family member RelB. J Biol Chem.

[70] Hecht E, Zago M, Sarill M, Rico de Souza A, Gomez A, Matthews J, Hamid Q, Eidelman DH, Baglole CJ (2014). Aryl hydrocarbon receptor-dependent regulation of miR-196a expression controls lung fibroblast apoptosis but not proliferation. Toxicol Appl Pharmacol.

[71] Sarill M, Zago M, Sheridan JA, Nair P, Matthews J, Gomez A, Roussel L, Rousseau S, Hamid Q, Eidelman DH, Baglole CJ (2015). The aryl hydrocarbon receptor suppresses cigarette-smoke-induced oxidative stress in association with dioxin response element (DRE)-independent regulation of sulfiredoxin 1. Free Radic Biol Med.

[72] Kamal A, Cincinelli A, Martellini T, Malik R (2015). A review of PAH exposure from the combustion of biomass fuel and their less surveyed effect on the blood parameters. Environ Sci Pollut Res Int.

[73] Durant JL, Busby WF, Lafleur AL, Penman BW, Crespi CL (1996). Human cell mutagenicity of oxygenated, nitrated and unsubstituted polycyclic aromatic hydrocarbons associated with urban aerosols. Mutat Res-Gen Tox En.

[74] Marcon A, Fracasso ME, Marchetti P, Doria D, Girardi P, Guarda L, Pesce G, Pironi V, Ricci P, de Marco R (2014). Outdoor formaldehyde and NO2 exposures and markers of genotoxicity in children living near chipboard industries. Environ Health Perspect.

[75] Placencia F, Fadic X, Yáñez K, Cereceda-Balic F (2019). Tradescantia as a biomonitor for genotoxicity evaluation of diesel and biodiesel exhaust emissions. Sci. Total environ. 651, no. Pt.

[76] Poma A, Colafarina S, Aruffo E, Zarivi O, Bonfigli A, Di Bucchianico S, Di Carlo P (2017). Effects of ozone exposure on human epithelial adenocarcinoma and normal fibroblasts cells. PLoS One.

[77] Karlsson HL, Ljungman AG, Lindbom J, Möller L (2006). Comparison of genotoxic and inflammatory effects of particles generated by wood combustion, a road simulator and collected from street and subway. Toxicol Lett.

[78] Nordin EZ, Uski O, Nyström R, Jalava P, Eriksson AC, Genberg J, Roldin P, Bergvall C, Westerholm R, Jokiniemi J, Pagels J, Boman C, Hirvonen M (2015). Influence of ozone initiated processing on the toxicity of aerosol particles from small scale wood combustion. Atmos. Environ.

[79] Zou LY, Zhang W, Atkiston S (2003). The characterisation of polycyclic aromatic hydrocarbons emissions from burning of different firewood species in Australia. Environ Pollut.

[80] Traboulsi H, Guerrina N, Iu M, Maysinger D, Ariya P, Baglole CJ (2017). Inhaled pollutants: the molecular scene behind respiratory and systemic diseases associated with ultrafine particulate matter. Int J Mol.

[81] Kjällstrand J, Petersson G (2001). Phenolic antioxidants in wood smoke. Sci Total Environ.

[82] Happo MS, Sippula O, Jalava PI, Rintala H, Leskinen A, Komppula M, Kuuspalo K, Mikkonen S, Lehtinen K, Jokiniemi J, Hirvonen M (2014). Role of microbial and chemical composition in toxicological properties of indoor and outdoor air particulate matter. Part Fibre Toxicol.

[83] Rice TM, Clarke RW, Godleski JJ, Al-Mutairi E, Jiang N, Hauser R, Paulauskis JD (2001). Differential ability of transition metals to induce pulmonary inflammation. Toxicol Appl Pharmacol.

[84] Riley MR, Boesewetter DE, Turner RA, Kim AM, Collier JM, Hamilton A (2005). Comparison of the sensitivity of three lung derived cell lines to metals from combustion derived particulate matter. Toxicol in Vitro.

[85] Uski O, Jalava PI, Happo MS, Torvela T, Leskinen J, Mäki-Paakkanen J, Tissari J, Sippula O, Lamberg H, Jokiniemi J, Hirvonen M.-. (2015). Effect of fuel zinc content on toxicological responses of particulate matter from pellet combustion in vitro. Sci Total Environ.

[86] Dasari S, Ganjayi MS, Yellanurkonda P, Basha S, Meriga B (2018). Role of glutathione S-transferases in detoxification of a polycyclic aromatic hydrocarbon, methylcholanthrene. Chem Biol Interact.

[87] Li Q, Lauer FT, Liu KJ, Hudson LG, Burchiel SW (2010). Low-dose synergistic immunosuppression of T-dependent antibody responses by polycyclic aromatic hydrocarbons and arsenic in C57BL/6J murine spleen cells. Toxicol Appl Pharmacol.

[88] Bakand S, Winder C, Hayes A (2007). Comparative in vitro cytotoxicity assessment of selected gaseous compounds in human alveolar epithelial cells. Toxicol in Vitro.

[89] Gamon LF, Wille U (2016). Oxidative damage of biomolecules by the environmental pollutants NO 2 • and NO 3. Acc Chem Res.

[90] Øvrevik J, Refsnes M, Låg M, Holme JA, Schwarze PE (2015). Activation of Proinflammatory responses in cells of the airway mucosa by particulate matter: oxidant- and non-oxidant-mediated triggering mechanisms. Biomolecules..

[91] Barrett EG, Henson RD, Seilkop SK, McDonald JD, Reed MD (2006). Effects of hardwood smoke exposure on allergic airway inflammation in mice. Inhal Toxicol.

[92] Gibbs-Flournoy EA, Gilmour MI, Higuchi M, Jetter J, George I, Copeland L, Harrison R, Moser VC, Dye JA (2018). Differential exposure and acute health impacts of inhaled solid-fuel emissions from rudimentary and advanced cookstoves in female CD-1 mice. Environ Res.

[93] Reed MD, Campen MJ, Gigliotti AP, Harrod KS, McDonald JD, Seagrave JC, Mauderly JL, Seilkop SK (2006). Health effects of subchronic exposure to environmental levels of hardwood smoke. Inhal Toxicol.

[94] Ward JE, Tan X (2007). Peroxisome proliferator activated receptor ligands as regulators of airway inflammation and remodelling in chronic lung disease. PPAR Res.

[95] Weng CM, Wang CH, Lee MJ, He JR, Huang HY, Chao MW, Chung KF, Kuo HP (2018). Aryl hydrocarbon receptor activation by diesel exhaust particles mediates epithelium-derived cytokines expression in severe allergic asthma. Allergy..

[96] Taddei A, Hediger F, Neumann FR, Gasser SM (2004). The function of nuclear architecture: A genetic approach. Annu Rev Genet.

[97] DFG (2002). Polycyclic aromatic hydrocarbons (PAH) [MAK value documentation, 2012]. The MAK-collection for occupational health and safety, Wiley-VCH Verlag GmbH & Co. KGaA.

[98] O'Driscoll CA, Gallo ME, Hoffmann EJ, Fechner JH, Schauer JJ, Bradfield CA, Mezrich JD (2018). Polycyclic aromatic hydrocarbons present in ambient urban dust drive proinflammatory T cell and dendritic cell responses via the aryl hydrocarbon receptor. PLoS One.

