# Supplementary material for: Influence of wood species on toxicity of log-wood stove combustion aerosols: a parallel animal and air-liquid interface cell exposure study on spruce and pine smoke
Source: Part Fibre Toxicol. 2020 Jun 15;17:27. doi: 10.1186/s12989-020-00355-1 (PMC7296712; doi:10.1186/s12989-020-00355-1)
Supplement: Supplementary file 1 — Additional file 1: Table S1. Calibration and quantification mixtures of isotope labelled internal standards and calibration standards. [file 12989_2020_355_MOESM1_ESM.pdf]

Supplementary Table 1: Calibration and quantification mixtures of isotope labelled internal standards and calibration standards.

Isotope labelled internal standard mixture	Calibration standard mixture
9,10-Anthracenedione 13C6	1(2H)-Acenaphthylenone
Acenaphthene D10	1,2,4-Trimethylanthracene
Acenaphthylene D8	1,2-Dimethylnaphthalene
Anthracene D10	1,3,6,8-Tetramethylpyrene
Benz[a]anthracene D12	1,3,7-Trimethylnaphthalene
Benz[a]pyrene D12	1,4-Naphthoquinone
Benz[e]pyrene D12	1,8-Naphthalic anhydride
Benzo[a]anthracene-7,12-dione D10	11H-Benzo[a]fluoren-11-one
Benzo[b]fluoranthene D12	11H-Benzo[b]fluoren-11-one
Benzo[bfluoranthene D12	1-Ethylpyrene
Benzo[ghi]perylene D12	1-Methyl-benz[a]anthracene
Benzo[k]fluoranthene D12	1-Methylfluorene
Benzo[k]fluoranthene D12	1-Methylpyrene
Biphenyl D10	1-Naphthaldehyde
Chrysene D12	2,2'-Binaphthalene
Coronene D12	2,3,6,7-Tetramethylanthracene
Dibenz[ah]anthracene D14	2,6-Dimethoxybenzoquinone
Dibenzothiophene D8	2-Phenylnaphthalene
Docosane D46	3,6-Dimethylphenanthrene
Eicosane D42	3-Methylfluorene
Fluoranthene D10	4,5-Dimethylpyrene
Fluorene D10	5,12-Naphthacenedione
Hexadecane D34	6-Ethylchrysene
Indeno[1,2,3-cd]pyrene D12	7,12-Dimethyl-Benz[a]anthracene
Naphthalene D8	9,10-Anthracenedione
Octadecane D38	9,10-Dimethylphenanthrene
Perylene D12	9H-Fluoren-9-one
Phenanthrene D10	9-Methylphenanthrene
Pyrene D10	Acenaphthene
Tetracosane D50	Acenaphthonquinone
Triacontane D62	Acenaphthylene
	Anthracene
4-Nitrophenol D4	Benz[a]anthracene
Adipic acid D10	Benz[a]pyrene
Cholesterol D6	Benz[e]pyrene
Dodecanol D25	Benzo[a]anthracene-7,12-dione
Fumaric acid D2	Benzo[b]fluoranthene
Glucose 13C6	Benzo[b]fluoranthene
Glycerol D8	Benzo[b]naphtho[1,2-d]thiophene
Levoglucosan 13C6	Benzo[b]naphtho[2,1-d]furan
Palmitic acid D31	Benzo[b]naphtho[2,1-d]thiophene
Vanillin 13C6	Benzo[b]naphtho[2,3-d]furan
	Benzo[c]phenanthrene
	Benzo[ghi]perylene
	Benzo[k]fluoranthene
	Benzo[k]fluoranthene
	Biphenyl
	Chrysene
	Coronene
	Cyclopenta(def)phenanthrenone
	Dibenz[ac]anthracene
	Dibenz[ah]anthracene
	Dibenzothiophene
	Fluoranthene
	Fluorene
	Indeno[1,2,3-cd]pyrene
	Methyleicosanoate
	n-Alkanes C12-C40
	Naphthalene
	Naphtho[2,1,8,7-klmn]xanthene
	Perylene
	Phenanthrene
	Picene

Pyrene
Retene
Xanthone

1-Hydroxypyrene
2-Hydroxy-1-naphthaldehyde
2-Ketoglutaric acid
2-Methyl-4-Nitrophenol
2-Methylerythritol
2-Naphthanol
3-Hydroglutaric acid
3-Hydroxyphenanthrene
4-Hydroxyphenanthrene
4-Nitrocatechol
4-Nitrophenol
9-Hydroxyphenanthrene
Abietic acid
Acetosyringone
Adipic Acid
Azelaic acid
Butanedioic acid
Cholesterol
Coniferaldehyde
Ergosterol
Erythritol
Fumaric acid
Galactosan
Glutaric acid
Hexanedioic acid
Hexanoic acid
Isopimaric acid
Levoglucosan
Linoleic acid
Maleic acid
Malic acid
Malonic acid
Mannosan
Methyl vanillate
Oleic acid
Palmitic acid
Phthalic acid
Salicylic acid
Sorbit
Stearic acid
Syringic Acid
Tartaric acid
Threitol
Trimellitic acid
Vanillic acid
Vanillin
