# Supplementary material for: Influence of wood species on toxicity of log-wood stove combustion aerosols: a parallel animal and air-liquid interface cell exposure study on spruce and pine smoke
Source: Part Fibre Toxicol. 2020 Jun 15;17:27. doi: 10.1186/s12989-020-00355-1 (PMC7296712; doi:10.1186/s12989-020-00355-1)
Supplement: Supplementary file 2 — Additional file 2. Supplementary materials and methods [file 12989_2020_355_MOESM2_ESM.pdf]

Supplementary materials and methods

Comet assay

Frozen cells (-80℃) were centrifuged (800g, 5min, +4℃) and most of the supernatant were discarded. Cell pellet was mixed and added to 80µl of low melting point agar (VWR, #444152G) and cell suspension was then transferred onto microscope slide, which agar (Amresco, #0710-100g) had been solidified in advanced, and spread with cover glass. After LMPA had solidified, the cover glass was removed and slides were submerged with decomposition buffer (75% NaCl, 19% Na_2EDTA, 0.6% Trizma base, 4% NaOH at H_2O, pH 10) for hour at +4℃. Decomposition buffer was then removed and neutralizing buffer (0.4M Tris) was applied on the slides. The slides were moved to electrophoresis chamber (VWR, KuroGEL Maxi 20) in which slides were allowed to balance for 40min in the dark. After balancing the electrophoresis was applied for 20min at 24V and 300mA (Bio-RAD, Power Pac 200). The slides were washed with neutralizing buffer and fixed during 10min incubation in 99% ethanol. The fixed slides were stained with ethidium bromide. From each sample 100 cells were analysed using the Comet assay IV software (Instem, UK). Experiment was controlled using a positive control prepared exposing A549 cells with methyl methanesulfonate (MMS) and mice exposed to diesel exhaust. The results are reported as median of the percentage of DNA in tail.

Reactive oxygen species (ROS)

ROS formation was determined using H_2DCFDA assay. H_2DCFDA levels was measured at three different timepoints, 0 min, 30 min and 60 min after H_2DCFDA reagent loading. Measurements was done by seeding cells with HBSS buffer into 96-well plate. First measurement was done immediately after loading 1µM H_2DCFDA onto cells with VICTOR3™ multilabel plate reader (model 1420-051, PerkinElmer, USA) at 492/530 nm. Between measurements the plate was incubated at +37°C and 5 % CO_2 and measurements were done 30 and 60 minutes after initial H_2DCFDA loading. Statistical analysis used as a parameter area under the curve.

Transcriptome and proteome analysis

RNA isolation

Total RNA of A549 and RAW264.7 cells was extracted using RNeasy Mini Plus Kit and total RNA from BALF was extracted using RNeasy Micro Kit (Qiagen, Venlo, The Netherlands). The Agilent 2100 Bioanalyzer was used to assess RNA quality and only high-quality RNA (RIN>7) was used for microarray analysis.

Microarray transcriptome profiling

Total RNA (about 30ng) was amplified using the Ovation Pico WTA System V2 in combination with the Encore Biotin Module (NuGEN Technologies, Inc, San Carlos, CA, USA). Amplified cDNA, from Raw264.7 macrophages and BALF, was hybridized on mouse Gene 2.1 ST arrays (Affymetrix, Santa Clara, CA, USA). Staining and scanning (Scanner 3000 7G) was done according to the Affymetrix expression protocol including minor modifications as suggested in the Encore Biotion protocol (NuGEN Technologies, Inc, San Carlos, CA, USA). A549 cells derived RNA was processed as previously reported by Kanashova and co-authors (Kanashova et al. 2018), Agilent Sure Print G3 Human Gene Expression Microarray 8 x 60K slides were used.

Transcriptome data analysis

Transcriptome Analysis Console (TAC; version 4.0.0.25; Thermo Fisher Scientific) was used for quality control and to obtain annotated normalized RMA gene-level data (standard settings including median polish and sketch-quantile normalization). Statistical analyses were performed by utilizing the statistical programming environment R. Downstream analyses were generated using QIAGEN’s Ingenuity Pathway Analysis (IPA®, QIAGEN Redwood City, www.qiagen.com/ingenuity).

In-solution digest

Proteome extraction was performed as previously described in Sapcariu et al. 2014. 40 µg of the RAW264.7 protein extracts were reduced with 1mM tris(2-carboxyethyl)phosphine (TCEP) and free sulfhydryl groups carbamidomethylated using 5.5mM choloroacetamide. The proteins were digested with sequencing grade endopeptidase LysC (Wako) in an enzyme to substrate ratio 1:50 for 3h at room temperature and subsequently diluted with four volumes of 50mM ammonium-bicarbonate. Tryptic digestion occurred for 10h at room temperature using sequencing grade trypsin (Promega) in an enzyme to substrate ratio 1:50. The reaction was stopped by adding trifluoroacetic acid (TFA) to a final concentration of 1% resulting in a final pH of 2. The peptides were purified by using C18 stage-tips (3M) according to (Rappsilber et al. 2003).

Dimethyl labeling

The eluted peptides were lyophilized in a speed-vac and reconstituted with 100µL 100 mM tri-ethyl ammonium bicarbonate (TEAB). The dimethyl labelling was performed in-solution adding 4µL light (+28Da), intermediate (+32Da, CD2O) or heavy formaldehyde (+36Da, 13CD2O). The final concentration was 0.15% formaldehyde in the presence of 0.8% sodium cyanoborohydride. The reaction was carried out overnight and quenched by adding 16µL ABC buffer and acidified by adding 8µl 50% TFA. The labelled samples were mixed with a 1:1:1 ratio and purified using stage-tips.

LC-MS/MS

For the proteomics analysis the peptides were separated using a 360min gradient (4 to 76% acetonitrile of 0.1% formic acid in water) at a flow rate of 0.3µL min^{-1} on a 0.1 x 200mm MonoCap C18 HighResolution Ultra column (GL Sciences, Japan). The separated peptides were ionized on a proxeon ion source and directly sprayed into the mass spectrometer (Q-Exactive Plus, Thermo). The MS1 acquisition was performed at a resolution of 70,000 in the scan range from 300 to 1700m z^{-1}

with automated gain control (AGC) target value of 1 × 106 and maximum injection time of 20ms. The top 10 intense masses were selected for MS2 analysis. MS2 scans were carried out at a resolution of 17,500 with the isolation window of 2.0m z^{-1}. Dynamic exclusion was set to 30s and the normalized collision energy to 26eV.

Proteome data analysis

For the automatic interpretation of the recorded spectral data, the MaxQuant software package version 1.5.2.8 was employed, using a multiplicity of 3 for dimethylation analysis (modified ε-amino groups of lysine plus modified N-terminal amino groups) (Cox and Mann 2008). Carbamidomethylation was set as a fixed modification while oxidized methionine and acetylated N-termini were set as variable modifications. An FDR of 1% was applied on peptide and protein level and an Andromeda-based search was performed using a mouse Uniprot database (uniprot.mouse.201805.fasta). The normalized medium/light or heavy/light ratios of the protein groups output file were used to determine proteins undergoing regulation.

Supplementary results

Oxidative stress

ROS formation was detected from A549 cells exposed at the Tox-ALI using H_2DCFDA oxidation from three different time points (supplementary Figure 5, additional file 11). DCF measurement revealed no increases in ROS levels after exposure to any of the studied emissions and altogether the measured H_2DCFDA oxidation was at the same level or decreased in all samples compared to controls.

References

Cox, J. & Mann, M. 2008. MaxQuant enables high peptide identification rates, individualized p.p.b.-range mass accuracies and proteome-wide protein quantification. Nat. Biotechnol. 26, 1367-1372.

Kanashova, T., Sippula, O., Oeder, S., Streibel, T., Passig, J., Czech, H., Kaoma, T., Sapcariu, S.C., Dilger, M., Paur, H., Sclager, C., Mulhopt, S., Weiss, C., Chmidt-Weber, C., Traidl-Hoffmann, C., Michlke, B., Krebs, T., Karg, E., Jakobi, G., Scholtes, S., Schnelle-Kreis, J., Sklorz, M., Orasche, J., Muller, L., Reda, A., Ruger, C., Neumann, A., Abbaszade, G., Radischat, C., Hiller, K., Kortelainen, M., Kuuspalo, K., Lamberg, H., Leskinen, J., Nuutinen, I., Torvela, T., Tissari, J., Jalava, P., Kasurinen, S., Uski, O., Hirvonen, M., Buters, J., Dittmar, G., Jokiniemi, J.K. & Zimmermann, R. 2018. Emissions from a modern log wood masonry heater and wood pellet boiler: Composition and biological impact on air-liquid interface exposed human lung cancer cells. J. Mol. Clin. Med. 1, 22-35. https://doi.org/10.31083/j.jmcm.2018.01.004

Rappsilber, J., Ishihama, Y. & Mann, M. 2003. Stop and go extraction tips for matrix-assisted laser desorption/ionization, nanoelectrospray, and LC/MS sample pretreatment in proteomics. Anal. Chem. 75, 663-670.
