# Supplementary material for: Influence of wood species on toxicity of log-wood stove combustion aerosols: a parallel animal and air-liquid interface cell exposure study on spruce and pine smoke
Source: Part Fibre Toxicol. 2020 Jun 15;17:27. doi: 10.1186/s12989-020-00355-1 (PMC7296712; doi:10.1186/s12989-020-00355-1)
Supplement: Supplementary file 3 — Additional file 3 Figure S1. Physical properties in the diluted exposure exhaust per day to day, for spruce (A) and pine (B) experiments. Parts A and C show average of 3 experiment days total suspended particulate mass (TSP), lung deposited surface area (LDSA) and particle number concentration (PNC) during exposures. [file 12989_2020_355_MOESM3_ESM.pdf]

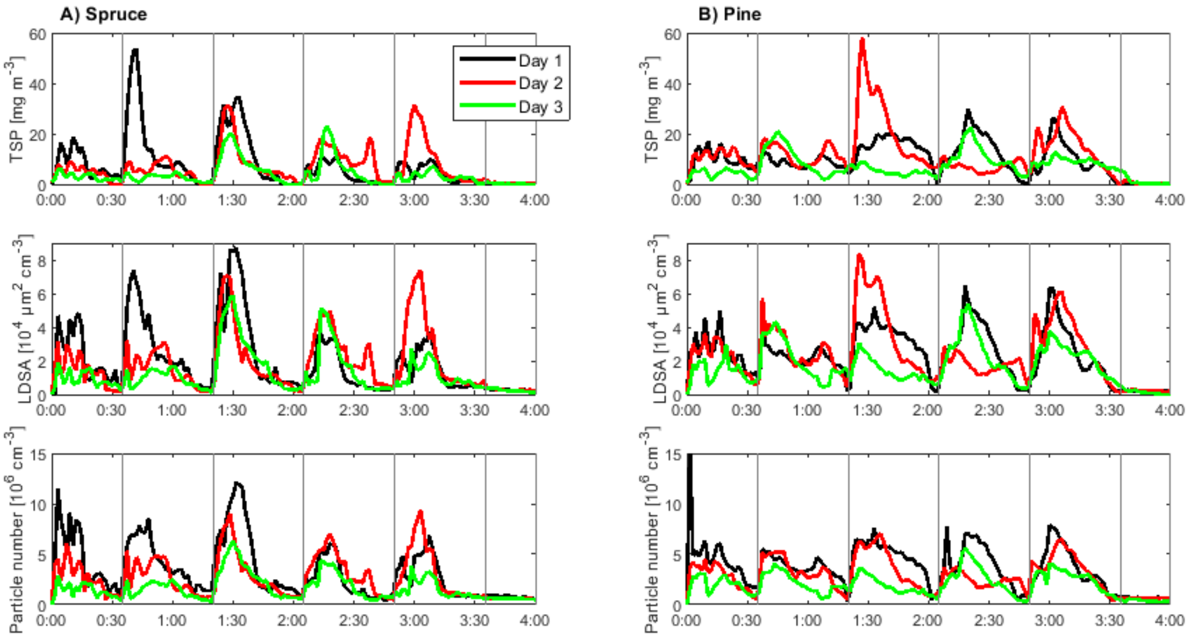

Supplementary Figure 1. Physical properties in the diluted exposure exhaust per day to day, for spruce (A) and pine (B) experiments. Parts A and C show average of 3 experiment days total suspended particulate mass (TSP), lung deposited surface area (LDSA) and particle number concentration (PNC) during exposures.
