# Supplementary material for: Influence of wood species on toxicity of log-wood stove combustion aerosols: a parallel animal and air-liquid interface cell exposure study on spruce and pine smoke
Source: Part Fibre Toxicol. 2020 Jun 15;17:27. doi: 10.1186/s12989-020-00355-1 (PMC7296712; doi:10.1186/s12989-020-00355-1)
Supplement: Supplementary file 4 — Additional file 4 Figure S2. Size of the particles measured by SMPS per day to day. The geometric mean diameters are shown with the solid lines, while GSDs are represented with dashed lines. (average of 3 experiments) [file 12989_2020_355_MOESM4_ESM.pdf]

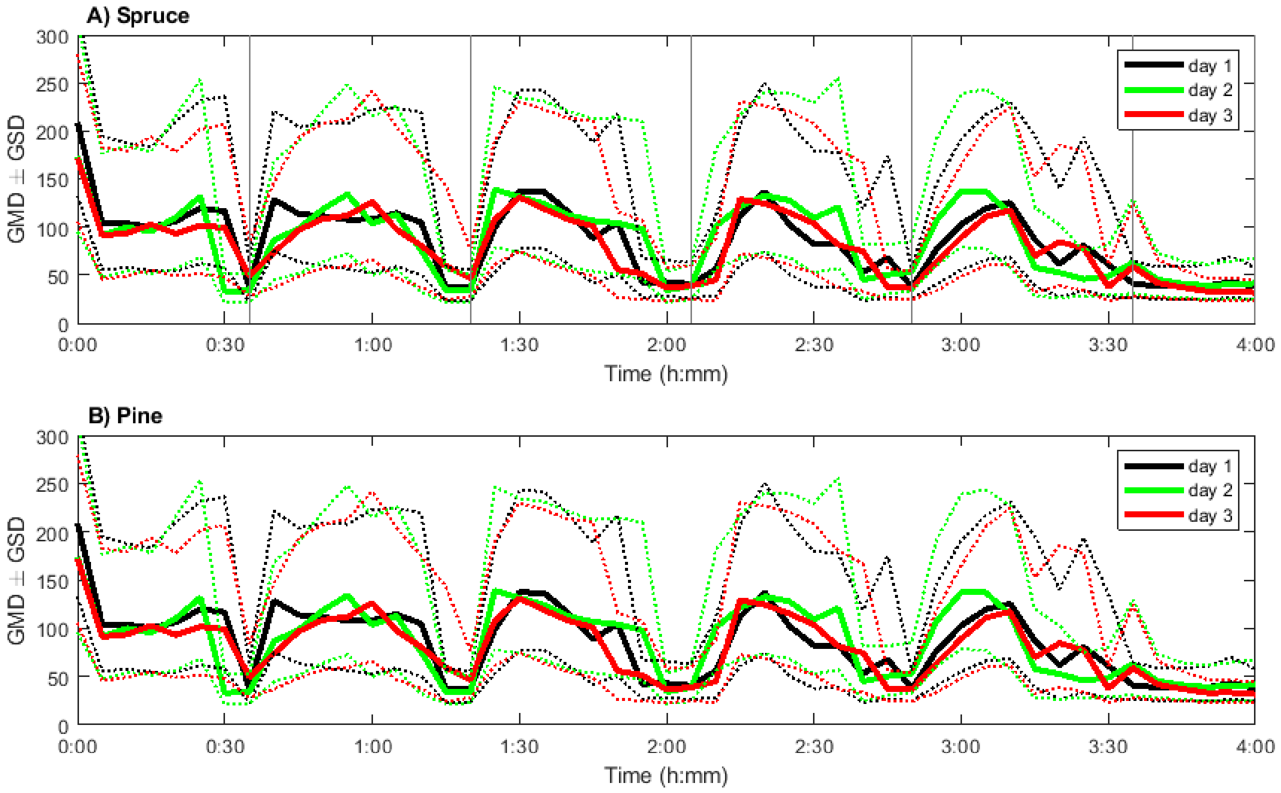

Supplementary Figure 2. Size of the particles measured by SMPS per day to day. The geometric mean diameters are shown with the solid lines, while GSDs are represented with dashed lines. (average of 3 experiments)
