# Supplementary material for: Influence of wood species on toxicity of log-wood stove combustion aerosols: a parallel animal and air-liquid interface cell exposure study on spruce and pine smoke
Source: Part Fibre Toxicol. 2020 Jun 15;17:27. doi: 10.1186/s12989-020-00355-1 (PMC7296712; doi:10.1186/s12989-020-00355-1)
Supplement: Supplementary file 6 — Additional file 6 Table S2. All measured organic compounds from wood combustion emissions. Organic compounds were sampled over 4-h exposure. Concentrations are calculated as averages from three test cycles ± standard deviation. [file 12989_2020_355_MOESM6_ESM.pdf]

Supplementary Table 2. All measured organic compounds from wood combustion emissions. Organic compounds were sampled over 4-hour exposure. Concentrations are calculated as averages from three test cycles ± standard deviation.

| Parameter | TEF | spruce (µg m-³) | TEQ | pine (µg m-³) | TEQ |
|---|---|---|---|---|---|
| ***PAH*** | | | | | |
| Phenanthrene | 0.001 | 4.4 ± 2.5 | 0.0044 | 23 ± 5 | 0.023 |
| Anthracene | | 1.2 ± 0.69 | | 6.2 ± 2.1 | |
| Fluoranthene | | 12 ± 3.5 | | 40 ± 5.4 | |
| Acephenanthrylene | | 5.3 ± 2 | | 16 ± 4.8 | |
| Pyrene | 0.001 | 11 ± 2.9 | 0.011 | 40 ± 8.8 | 0.04 |
| Benzo[c]phenanthrene | | 0.85 ± 0.35 | | 2.9 ± 1.1 | |
| Benzo[ghi]fluoranthene | | 4.5 ± 1.3 | | 16 ± 3.4 | |
| Benz[a]anthracene | 0.1 | 2.9 ± 1 | 0.29 | 8.4 ± 2.4 | 0.84 |
| Cyclopenta[cd]pyrene | 0.1 | 4.5 ± 3.1 | 0.45 | 21 ± 12 | 2.1 |
| Chrysene | 0.01 | 4.5 ± 1.8 | 0.045 | 15 ± 5.5 | 0.15 |
| sum_Benzo[b,k]fluoranthene | 0.1 | 7.2 ± 2.6 | 0.72 | 25 ± 7.1 | 2.5 |
| 2,2'-Binaphthalene | | 0.36 ± 0.13 | | 0.9 ± 0.42 | |
| Benzo[e]pyrene | | 5.1 ± 1.7 | | 16 ± 6.8 | |
| Benzo[a]pyrene | 1 | 2.1 ± 0.79 | 2.1 | 6.6 ± 3.4 | 6.6 |
| Perylene | | 0.54 ± 0.18 | | 2.1 ± 1.4 | |
| Anthanthrene | 0.1 | 0.14 ± 0.046 | 0.014 | 0.48 ± 0.23 | 0.048 |
| Dibenz[ah]anthracene | 1 | 0.13 ± 0.039 | 0.13 | 0.42 ± 0.043 | 0.42 |
| Indeno[1,2,3-cd]pyrene | 0.1 | 1.1 ± 0.39 | 0.11 | 3.4 ± 1.4 | 0.34 |
| Picene | | 0.24 ± 0.11 | | 0.67 ± 0.5 | |
| Benzo[ghi]perylene | | 2.2 ± 0.75 | | 7.7 ± 3.3 | |
| Coronene | | 1.1 ± 0.49 | | 3.6 ± 2 | |
| Naphtho[1,2-b]fluoranthene | | 1.4 ± 0.65 | | 4.2 ± 2.3 | |

| | | | | | | | | | |
|---|---|---|---|---|---|---|---|---|---|
| Dibenzo[al]pyrene | 10 | 1 | ± | 0.48 | 10 | 2.7 | ± | 1.4 | 27 |
| Naphtho[2,3-b]fluoranthene | | 1 | ± | 0.54 | | 3.3 | ± | 2.2 | |
| Dibenzo[ae]pyrene | 1 | 0.33 | ± | 0.21 | 0.33 | 0.76 | ± | 0.67 | 0.76 |
| Naphtho[2,1-a]pyrene | | 0.56 | ± | 0.22 | | 1.1 | ± | 0.7 | |
| Naphtho[2,3-a]pyrene | | 0.4 | ± | 0.25 | | 1.9 | ± | 0.94 | |
| Dibenzo[ai]pyrene | 10 | 0.13 | ± | 0.06 | 1.3 | 0.36 | ± | 0.37 | 3.6 |
| Dibenzo[ah]pyrene | 10 | 0.091 | ± | 0.051 | 0.91 | 0.2 | ± | 0.21 | 2 |
| ***Alkylated PAH*** | | | | | | | | | |
| 9-Methylphenanthrene | | 0.29 | ± | 0.42 | | 0.41 | ± | 0.58 | |
| 3,6-Dimethylphenanthrene | | 0.005 | ± | 0.008 | | 0.006 | ± | 0.009 | |
| Retene | | 0.26 | ± | 0.21 | | 0.61 | ± | 0.03 | |
| 7,12-Dimethyl-Benz[a]anthracene | | 0,14 | ± | 0.075 | | 0.21 | ± | 0.3 | |
| 1-Methyl-benz[a]anthracene | | 0.018 | ± | 0.011 | | 0.033 | ± | 0.047 | |
| ***o-PAH*** | | | | | | | | | |
| 9H-Fluoren-9-one | | 12 | ± | 6.6 | | 38 | ± | 27 | |
| 1H-Phenalen-1-one | | 85 | ± | 22 | | 180 | ± | 130 | |
| Cyclopenta(def)phenanthrenone | | 5.6 | ± | 2.5 | | 22 | ± | 7.5 | |
| 11H-Benzo[a]fluoren-11-one | | 1.5 | ± | 0.61 | | 5.3 | ± | 1.2 | |
| 11H-Benzo[c]fluoren-11-one | | 0.61 | ± | 0.29 | | 2 | ± | 0.66 | |
| 11H-Benzo[b]fluoren-11-one | | 2.2 | ± | 0.99 | | 6.5 | ± | 1.9 | |
| Xanthone | | 1.2 | ± | 0.37 | | 5.2 | ± | 0.83 | |
| 1,8-Dihydroxynaphthalene | | 0.44 | ± | 0.12 | | 2.6 | ± | 0.67 | |
| 1,5-Dihydroxynaphthalene | | 0.036 | ± | 0.027 | | 0.05 | ± | 0.071 | |
| 1,8-Naphthalic anhydride | | 13 | ± | 3.1 | | 52 | ± | 11 | |
| 9,10-Anthracenedione | | 3.2 | ± | 0.97 | | 13 | ± | 2.4 | |
| Benz[a]anthracene-7,12-dione | | 0.19 | ± | 0.059 | | 0.7 | ± | 0.14 | |

| | | |
|---|---|---|
| 5,12-Naphthacenedione | 0.35 ± 0.22 | 0.92 ± 0.45 |
| ***Anhydro-sugars*** | | |
| Galactosan | 0.095 ± 0.04 | 0.35 ± 0.15 |
| Mannosan | 0.28 ± 0.053 | 0.62 ± 0.13 |
| Levoglucosan | 3.5 ± 1.9 | 4.9 ± 3.6 |
| ***Resin acids*** | | |
| Isopimaric acid | 0.005 ± 0.006 | 0.003 ± 0.002 |
| Dehydroabietic acid, methyl ester | 0,029 ± 0.029 | 0.049 ± 0.007 |
| Dehydroabietic acid | 1.1 ± 1 | 1.4 ± 0.25 |
| Abietic acid | 0.012 ± 0.004 | 0.024 ± 0.006 |
