# Supplementary material for: Influence of wood species on toxicity of log-wood stove combustion aerosols: a parallel animal and air-liquid interface cell exposure study on spruce and pine smoke
Source: Part Fibre Toxicol. 2020 Jun 15;17:27. doi: 10.1186/s12989-020-00355-1 (PMC7296712; doi:10.1186/s12989-020-00355-1)
Supplement: Supplementary file 7 — Additional file 7 Table S3. Inorganic elements from spruce and pine combustion emissions. Organic compounds were sampled over 4-h exposure. Concentrations are calculated as averages from three test cycles ± standard deviation. [file 12989_2020_355_MOESM7_ESM.pdf]

Supplementary Table 3. Inorganic elements from spruce and pine combustion emissions. Organic compounds were sampled over 4-hour exposure. Concentrations are calculated as averages from three test cycles ± standard deviation.

		Spruce		Pine	
element	unit	Avg.	Sd	Avg.	Sd
Al	µg m^{-3}	1.37	0.18	1.02	0.83
As	ng m^{-3}	6.9	1.5	5.67	1.11
B	ng m^{-3}	80.7	47.6	136	87
Ba	ng m^{-3}	37.3	13.7	14.6	10.4
Be	ng m^{-3}	22.9	6.5	18.6	10.9
Ca	µg m^{-3}	3.47	0.41	3.95	0.52
Cd	ng m^{-3}	137	33	167	5
Co	ng m^{-3}	0.8	0.2	1.07	0.59
Cr	ng m^{-3}	57.3	16.5	45	22
Cu	ng m^{-3}	903	920	398	322
Fe	µg m^{-3}	1.51	0.91	0.667	0.377
Hg	pg m^{-3}	263	69	815	571
K	µg m^{-3}	124	27	75.1	31
Li	ng m^{-3}	5.53	1.51	3.22	0.34
Mg	µg m^{-3}	0.62	0.13	1.03	0.1
Mn	ng m^{-3}	153	15	113	22
Mo	ng m^{-3}	2.57	0.21	9.03	9.18
Na	µg m^{-3}	1.6	0.5	0.866	0.13
Ni	ng m^{-3}	27.3	8.1	24.2	13.4
Pb	ng m^{-3}	121	19	65.8	14.9
S	µg m^{-3}	17	4	8.56	2.75
Sb	ng m^{-3}	473	98	769	374

Se	ng m^{-3}	1.29	0.24	2.03	0.46
Sn	ng m^{-3}	65	21	14.4	3.8
Sr	ng m^{-3}	15.9	2.1	8.61	0.29
Ti	ng m^{-3}	29.7	6.9	37.9	20.9
V	ng m^{-3}	0.773	0.2	0.595	0.196
W	ng m^{-3}	186	12	88.4	16.5
Zn	µg m^{-3}	11.9	1.7	6.87	0.88
