# Supplementary material for: Influence of wood species on toxicity of log-wood stove combustion aerosols: a parallel animal and air-liquid interface cell exposure study on spruce and pine smoke
Source: Part Fibre Toxicol. 2020 Jun 15;17:27. doi: 10.1186/s12989-020-00355-1 (PMC7296712; doi:10.1186/s12989-020-00355-1)
Supplement: Supplementary file 8 — Additional file 8 Figure S4. Viability (A), Comet assay (B) and IL-8 secretion (C) of A549 control cells after exposed one hour at incubator with CO2, incubator without CO2 or Tox-ALI to clean air. Each bar shows mean ± SEM, n = 3. [file 12989_2020_355_MOESM8_ESM.pdf]

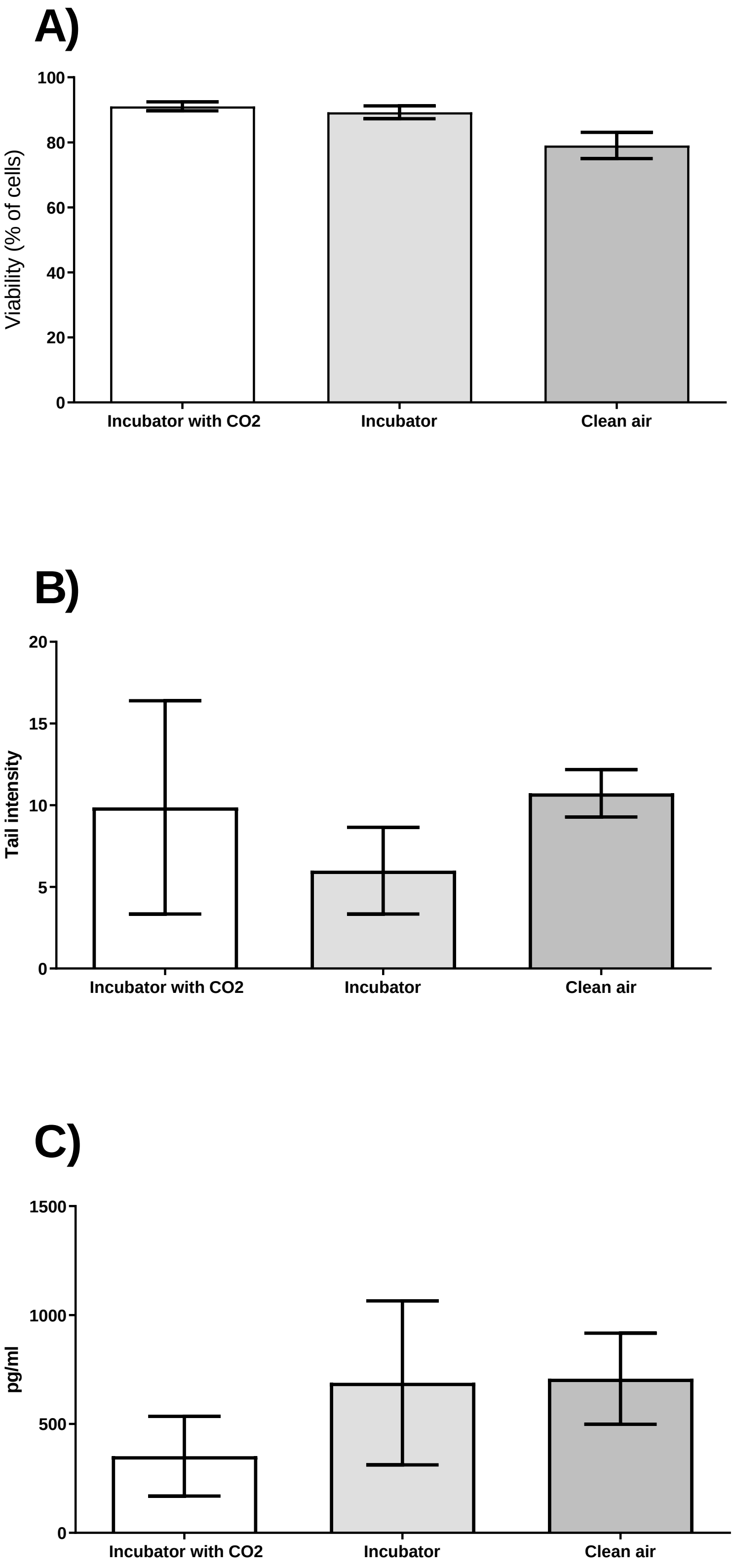

Supplementary Figure 4. Viability (A), Comet assay (B) and IL-8 secretion (C) of A549 control cells after exposed one hour at incubator with CO2, incubator without CO2 or Tox-ALI to clean air. Each bar shows mean ± SEM, n=3.
