# Supplementary material for: Influence of wood species on toxicity of log-wood stove combustion aerosols: a parallel animal and air-liquid interface cell exposure study on spruce and pine smoke
Source: Part Fibre Toxicol. 2020 Jun 15;17:27. doi: 10.1186/s12989-020-00355-1 (PMC7296712; doi:10.1186/s12989-020-00355-1)
Supplement: Supplementary file 9 — Additional file 9 Table S4. Top 5 significant ingenuity canonical pathways of spruce aerosol exposure. [file 12989_2020_355_MOESM9_ESM.pdf]

Supplementary Table 4. Top 5 significant ingenuity canonical pathways of spruce aerosol exposure.

A549 Spruce AS	-log(p-value)	Ratio	z-score
Role of Oct4 in Mammalian Embryonic Stem Cell Pluripotency	3.08	2.44E-01	
Role of PKR in Interferon Induction and Antiviral Response	2.84	2.44E-01	
Death Receptor Signaling	2.49	1.74E-01	-2.000
Hepatic Fibrosis / Hepatic Stellate Cell Activation	2.08	1.37E-01	
Bladder Cancer Signaling	2.06	1.61E-01	
A549 Spruce HV			
Role of PKR in Interferon Induction and Antiviral Response	3.22	2.68E-01	
Differential Regulation of Cytokine Production in Macrophages and T Helper Cells by IL-17A and IL-17F	2.48	3.33E-01	
Role of Oct4 in Mammalian Embryonic Stem Cell Pluripotency	2.32	2.22E-01	
AMPK Signaling	2.13	1.4E-01	
Cell Cycle Control of Chromosomal Replication	2.07	1.96E-01	
Raw264.7 Spruce AS			
IL-17A Signaling in Gastric Cells	3.2	2.73E-01	2.236
Role of CHK Proteins in Cell Cycle Checkpoint Control	2.77	1.64E-01	
GADD45 Signaling	2.66	2.63E-01	
IL-17A Signaling in Fibroblasts	2.3	1.88E-01	
Role of BRCA1 in DNA Damage Response	2.14	1.25E-01	
Raw264.7 Spruce HV			
Role of CHK Proteins in Cell Cycle Checkpoint Control	5.16	2.36E-01	
TNFR2 Signaling	4.64	0.3	
IL-7 Signaling Pathway	4.3	1.79E-01	
IL-17A Signaling in Gastric Cells	3.9	3.18E-01	2.236
IL-17A Signaling in Fibroblasts	3.57	2.5E-01	
BALF Spruce AS			
Epoxysqualene Biosynthesis	3.28	1	
Glutathione-mediated Detoxification	2.7	1.67E-01	2.000

Nicotine Degradation III	2.64	1.22E-01
TREM1 Signaling	2.31	8.7E-02
Nicotine Degradation II	2.26	0.1
