# Supplementary material for: Influence of wood species on toxicity of log-wood stove combustion aerosols: a parallel animal and air-liquid interface cell exposure study on spruce and pine smoke
Source: Part Fibre Toxicol. 2020 Jun 15;17:27. doi: 10.1186/s12989-020-00355-1 (PMC7296712; doi:10.1186/s12989-020-00355-1)
Supplement: Supplementary file 10 — Additional file 10 Table S5. Top 5 significant ingenuity canonical pathways of pine aerosol exposed cells. [file 12989_2020_355_MOESM10_ESM.pdf]

Supplementary Table 5. Top 5 significant ingenuity canonical pathways of pine aerosol exposed cells.

A549 Pine AS	-log(p-value)	Ratio	z-score
Protein Ubiquitination Pathway	3.26	1.44E-01	
Aryl Hydrocarbon Receptor Signaling	3.13	1.69E-01	
p38 MAPK Signaling	3.1	1.75E-01	-2.400
Aldosterone Signaling in Epithelial Cells	2.9	1.56E-01	-2.449
Role of PKR in Interferon Induction and Antiviral Response	2.82	2.44E-01	
A549 Pine HV			
Osteoarthritis Pathway	3.52	1.55E-01	
Aryl Hydrocarbon Receptor Signaling	3.21	1.69E-01	
Wnt/Ca+ pathway	2.9	2.1E-01	
Aldosterone Signaling in Epithelial Cells	2.66	1.5E-01	-2.449
Differential Regulation of Cytokine Production in Macrophages and T Helper Cells by IL-17A and IL-17F	2.64	3.33E-01	
Raw264.7 Pine AS			
NRF2-mediated Oxidative Stress Response	3.57	1.18E-01	
MIF Regulation of Innate Immunity	3.06	0.2	
Pancreatic Adenocarcinoma Signaling	2.95	1.27E-01	
iNOS Signaling	2.78	1.82E-01	
IL-10 Signaling	2.71	1.52E-01	
Raw264.7 Pine HV			
Role of Macrophages, Fibroblasts and Endothelial Cells in Rheumatoid Arthritis	4.62	1.15E-01	
Small Cell Lung Cancer Signaling	4.32	1.76E-01	
TNFR2 Signaling	3.83	2.67E-01	2.646
IL-10 Signaling	3.7	1.82E-01	
Acute Phase Response Signaling	3.65	1.28E-01	
BALF Pine AS			
Agranulocyte Adhesion and Diapedesis	3.73	6.67E-02	
Crosstalk between Dendritic Cells and Natural Killer Cells	2.85	8.7E-02	

Glucocorticoid Receptor Signaling	2.76	4.42E-02
Circadian Rhythm Signaling	2.57	1.21E-01
Communication between Innate and Adaptive Immune Cells	2.25	7.81E-02
