# Supplementary material for: Influence of wood species on toxicity of log-wood stove combustion aerosols: a parallel animal and air-liquid interface cell exposure study on spruce and pine smoke
Source: Part Fibre Toxicol. 2020 Jun 15;17:27. doi: 10.1186/s12989-020-00355-1 (PMC7296712; doi:10.1186/s12989-020-00355-1)
Supplement: Supplementary file 11 — Additional file 11 Figure S5. Area under curve (AUC) calculated from exposed A549 cells from three different timepoints of H2DCFDA measurements. Each bar shows mean ± SEM, n = 3. [file 12989_2020_355_MOESM11_ESM.pdf]

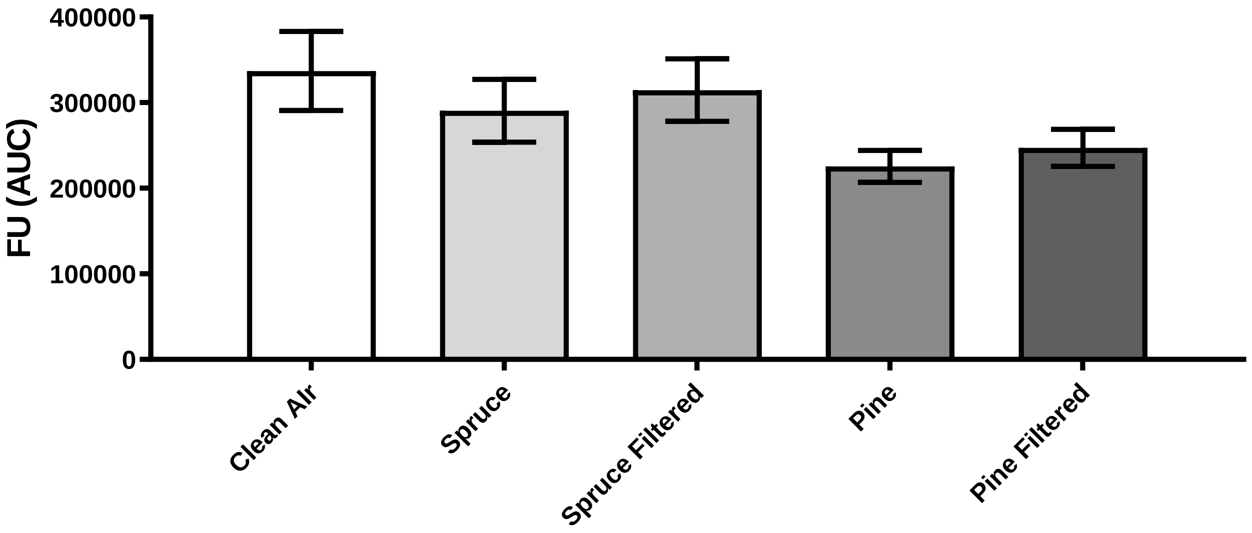

Supplementary Figure 5. Area under curve (AUC) calculated from exposed A549 cells from three different timepoints of H_2DCFDA measurements. Each bar shows mean ± SEM, n=3.
